# Identification of marginal zone B cells in head and neck cancer with immunomodulatory characteristics

**DOI:** 10.1016/j.jare.2025.11.070

**Published:** 2025-11-29

**Authors:** Jiantong Bao, Jochen Hess, Annika C. Betzler, Zhen Zhang, Miltiadis Tsesmelis, Sebastian Schmid, Gaurav Berry, Patrick J. Schuler, Jens Greve, Simon Laban, Thomas K. Hoffmann, Medhanie Mulaw, Cornelia Brunner

**Affiliations:** a Department of Otorhinolaryngology and Head & Neck Surgery, University Medical Center Ulm, Head & Neck Cancer Center of the Comprehensive Cancer Center Ulm, Ulm, Germany; b School of Medicine, Southeast University, Nanjing, China; c Department of Otorhinolaryngology, Head and Neck Surgery, Heidelberg University Hospital, Heidelberg, Germany; d Unit for Single-Cell Genomics, Medical Faculty, Ulm University, Ulm, Germany; e Department of Anesthesiology and Intensive Care Medicine, Ulm University Medical Center, Ulm, Germany; f Core Facility Immune Monitoring, Medical Faculty, Ulm University, Ulm, Germany

**Keywords:** Marginal zone B cells, Head and neck squamous cell carcinoma, Antigen presentation, MZB-Tfh-GCB axis

## Abstract

**Introduction:**

Recently we observed high numbers of marginal zone B cells (MZBs) within murine head and neck squamous cell carcinoma (HNSCC) with immunosuppressive potential. To date, MZBs have not been linked to tumor development or tumor prevention.

**Objectives:**

Based on our previous findings the present study aimed to validate the presence of MZB in HNSCC and to investigate their possible implications in tumorigenesis and prognosis.

**Methods:**

Flow cytometry was used to uncover MZB within tumors and blood of HNSCC patients. A single-cell RNA sequencing cohort of 118 HNSCC patients across different disease stages and 6 healthy donors (HDs) was compiled. Comparative transcriptomic profiling of B lymphocytes between HNSCC and HDs were performed. Downstream analysis, such as pathway enrichment, cell–cell communication, pseudotime trajectory inference, survival correlation, and spatial transcriptomics were applied.

**Results:**

Two MZB subsets were revealed in tissues and blood of HNSCC patients and HDs. The tumor-associated MZBs were featured with hypoxia stress and viral-related hallmark genes. MZB-2, characterized by elevated expression of activation markers and immune-regulatory genes, displayed strong interactions with CD4^+^ T cells and antigen-presenting cells. These interactions were supported by costimulatory signals in HDs but were absent in HNSCC patients. Co-localization of MZB-2, germinal center B cell (GCB), and CD4^+^ follicular helper T cell (Tfh) was detected in HNSCC, suggesting the presence of an intratumoral MZB-Tfh-GCB axis. Clinically, MZB-2 abundance was associated with favorable prognosis in early-stage HNSCC, but not in advanced disease. Immunosuppressive gene signatures were not exclusive to MZBs, indicating that they do not represent a purely regulatory B cell phenotype.

**Conclusion:**

Our findings demonstrate an immunomodulatory role of MZBs in tumor immunity, balancing antigen presentation, cytokine signaling, and immune suppression. The association of MZB-2 with improved prognosis in early-stage HNSCC highlights its potential as a beneficial regulator of antitumor immunity during early tumor progression.

## Highlights

• Two distinct marginal zone B cell (MZB) subsets were identified in head and neck squamous cell carcinoma (HNSCC).
• Tumor-associated MZBs were enriched in HPV^+^ HNSCC and characterized by hypoxia stress and viral-related hallmark genes.
• MZB-2 display strong MHC-I/II signaling and T cell interaction, with costimulatory signals observed in healthy donors but absent in HNSCC.
• MZB-2 crosstalk with CD4^+^ T and antigen-presenting cells, boost the immune system and are prognostic relevant at early-stage HNSCC.
• Colocalization of MZB-2, germinal center B cell (GCB), and CD4^+^ follicular helper T cell (Tfh) suggests a MZB–Tfh–GC axis in HNSCC.

## Graphical abstract

**Figure:**
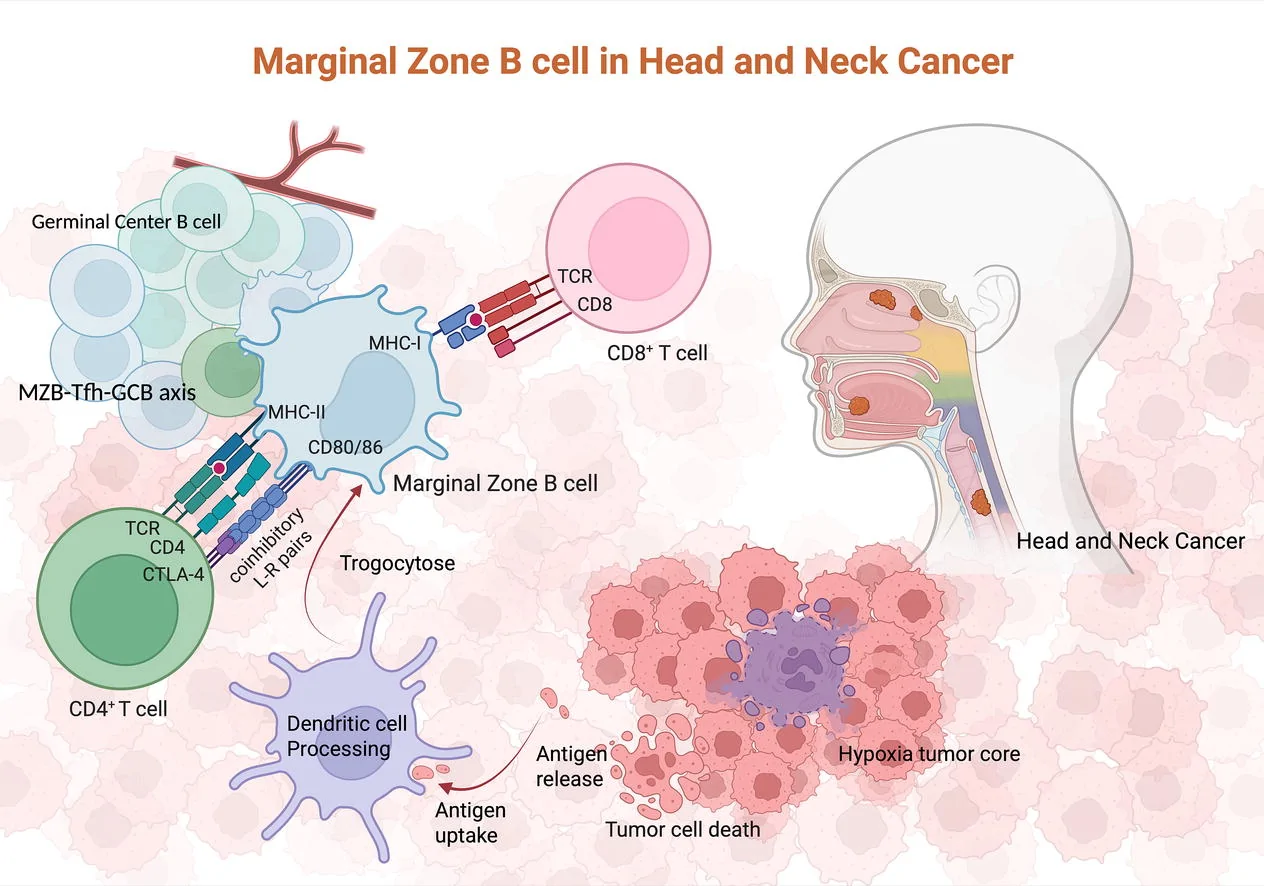

## Introduction

Head and neck squamous cell carcinoma (HNSCC) presents a unique challenge due to its dismal prognosis, characterized by profound psychological distress and compromised patients’ quality of life [1]. Immunotherapy, particularly immune checkpoint inhibition (ICI), has emerged as a promising treatment for recurrent, metastatic, or unresectable HNSCC. However, clinical responses to ICI are often variable, with many patients achieving only modest benefits, and a minority even experiencing accelerated disease progression, a phenomenon known as hyperprogression [2]. This underscores the need for a deeper understanding of the tumor immune microenvironment (TIME) to identify predictive biomarkers and improve therapeutic strategies.

While historically underappreciated, tumor-infiltrating B cells (TIBs) are now recognized as important cellular components of the TIME, capable of exerting both pro- and anti-tumor effects. In order to better understand the composition of different B-cell subpopulations in HNSCC, we recently investigated a murine HNSCC model and found a large number of marginal zone B cells (MZBs) in the TIME [3]. Among all B-cell subpopulations analyzed, MZBs most strongly expressed the ectonucleotidases CD39 and CD73, enzymes that convert extracellular ATP into immunosuppressive adenosine, suggesting a contribution of MZBs to the immunosuppressive tumor microenvironment (TME) in HNSCC [3].

Under physiological conditions, MZBs reside mainly in the marginal zone (MZ) of the spleen where blood is filtered and represent a first line of defense against foreign pathogens [4]. Equipped with pathogen-recognition receptors (PRRs) like Toll-like receptors (TLRs) and a polyreactive B-cell receptor (BCR), MZBs can quickly recognize invading pathogens and respond to them with rapid and high secretion of class M immunoglobulins (IgM), independently of T cell’s help. MZBs are therefore part of the innate arm of immunity. After capturing of an antigen, MZBs shuttle from the MZ to the follicles to deliver immune-complexed antigens to follicular-dendritic cells (FDCs) [5,6], essential for the formation of germinal centers (GC). Additionally, MZBs are capable to migrate into T-cell zones of the follicle to activate CD4^+^ T cells [7,8,9]. Therefore, MZB are also involved in T-cell-dependent immune responses.

Besides these immune-system activating functions, MZBs as well as T2-MZB precursor B cells (T2-MZP-B-cells) also exhibit immune-regulatory activities by secreting immunosuppressive IL-10 [10,11]. Moreover, a recently identified MZB precursor population (MZP) expresses IL-10, the immune checkpoint molecule PD-1 and the aforementioned ectonucleotidases CD39 and CD73 able to convert ATP to immunosuppressive adenosine [12,13]. MZBs have not yet been linked to cancer. However, the T2-MZP-B-cells in tumor-draining lymph nodes in B16-F10 melanoma-bearing mice have been found to have a tumor-promoting function in B-cell-deficient mice [14]. Thus, it is not clear whether MZBs revealed by us in the mouse model of HNSCC have pro- or anti-tumorigenic activity. The question became even more complex after two MZB subpopulations were identified in humans, MZB-1 and MZB-2, characterized by divergent transcriptomic profiles and developmental lineages [15]. Also in mice, two different MZB populations were identified based on their CD80 expression level, that differ in their autoreactivity, radiosensitivity, and functional capacity [16].

Therefore, the present study aimed to confirm the presence of MZB in human HNSCC, to reveal distinct MZB subpopulations and to explore their possible role in HNSCC pathogenesis and prognosis. Here, we demonstrate the presence of MZBs in both peripheral blood and the TME of HNSCC patients. Furthermore, based on the transcriptome profile, we were able to identify two distinct MZB populations. Contrary to our original assumption that MZBs contribute solely to the immunosuppressive TME, our results suggest that MZBs also participate in antigen presentation and immunomodulation, thereby enhancing anti-tumor immunity particularly in early-stage HNSCC. However, further *in vitro* and *in vivo* as well as clinical studies are required to completely understand the role of MZB in HNSCC tumorigenesis.

Knowledge about pro- or anti-tumorigenic MZBs in the TME of HNSCC contributes to a better understanding of immunological processes and could thus form the basis for the development of new and innovative immune-based strategies for the treatment of HNSCC patients.

## Materials and methods

### Study design

The study investigates the role of MZBs in HNSCC using flow cytometry, scRNA-seq and spatial transcriptome data. It identifies distinct MZB subsets, analyzes their interactions with other TILs in the tumor microenvironment, compares their functional roles in the tumor immune milieu versus normal tissues, evaluates their immunomodulatory potential, and explores their prognostic significance.

### Experimental subject details

Our study involved HNSCC patients and healthy volunteers in both genders. Sex was not considered as a biological variable in this study. Human tumor tissues and blood samples were used. Human tumor tissue was obtained during surgery from HNSCC patients; whereas blood samples were obtained from patients with HNSCC and healthy volunteers (non-tumor patients). The human papillomavirus (HPV) status of the tumor biopsies was determined by p16 immunohistochemistry and polymerase chain reaction (PCR) for HPV DNA. Detailed clinic-pathological data were summarized in **Tables S1-S9**.

Three panels were established for the detection of MZB, T2-MZP, and germinal center B cells (GCB) by flow cytometry (**Tables S10-S12**). The MZB cohort composed of 81 peripheral blood mononuclear cell (PBMC) and 24 tumor infiltrating lymphocyte (TIL) samples from HNSCC patients, and 20 PBMC samples from healthy donors (HD) for comparison (**Tables S1-S3**). The GCB cohort was composed of 42 PBMC and 16 TIL cases of HNSCC patients, with 15 HD PBMC for comparison (**Tables S4-S6**), whereas the T2-MZP cohort contained 38 PBMC and 20 TIL samples of HNSCC patients, and 10 HD PBMC samples (**Tables S7-S9**).

### PBMC isolation

PBMC were isolated from whole blood samples of patients medicated in the ENT Clinic, Ulm University Medical Center. Fresh PBMC were first isolated using a standard density gradient centrifugation method as described earlier [17].

### Tumor dissociation and TIL isolation

The tumor tissue was obtained and processed according to the tumor dissociation kit (Miltenyi Biotec) protocol and the genleMACS^TM^ tissue dissociator (Miltenyi Biotec) as described elsewhere [17]. CD45 (TIL) MicroBeads from Miltenyi Biotec were used for the magnetic purification of CD45^+^ TILs. The isolated CD45^+^ TILs were collected and directly used for flow cytometry analysis.

### Flow cytometry

Single-cell suspensions of PBMC and CD45^+^ TILs were used for flow cytometry applying standard staining protocols. The antibodies employed for the panels are detailed in the Key Resources Table in the Supplementary Material. Samples in the MZP cohort were first stained with Rhodamine 123 (R123) for 10 min, then washed and chased for 3 h before staining with other antibodies. Data acquisition was performed using a Gallios cytometer (Beckman Coulter, USA) and analyzed with Kaluza Analysis software, version 2.1 (Beckman Coulter, USA). All results were calculated using Excel (Microsoft Office 2019) and further processed with GraphPad Prism Software (version 10.0).

### Single-cell transcriptomics analysis

Four GSE datasets (GSE139324, GSE164690, GSE181919, GSE234933) of HNSCC patients and healthy volunteers were selected and integrated [[18], [19], [20], [21]]. Overall, 118 HNSCC patients (with samples from 86 primary HNSCC, 16 local recurrence, 4 lymph node (LN) metastasis, 12 distant metastasis), 9 normal *para*-carcinoma tissue, 6 tonsil samples of HD, and 4 samples of leukoplakia were accumulated. Using the Seurat R package (version 4.2.0), sorted CD45^+^ cells with mitochondrial transcripts above 15 %, ribosome transcripts above 3 %, number of unique genes per cell below 500, and total RNA transcripts per cell above 20,000 were filtered out. Genes only expressed in 3 cells or fewer in the entire dataset were filtered out. The data were normalized using *SCTransform* and integrated using *Harmony* (with sample ID as vector covariant) [22,23]. For more efficient clustering and dimension reduction, principal components analysis (PCA) was performed on the top 3000 transcriptomic variable features, and the top 15 principal components (PCs) were selected based on the Elbow plot and used for downstream analysis. Cells were first clustered using the *Seurat*::*FindClusters* function with the resolution set as 0.1 to separate major immune cell clusters. Then the B cell population was abstracted for the second round of subpopulation clustering (with 30 PCs and with cluster resolution of 1.3).

The annotation of B cell subclusters was performed using three complementary steps. First, the SingleR (version 1.10.0) package was utilized for reference-based scRNA-seq annotation [24]. SingleR uses reference transcriptomic datasets of known cell types to predict the identities of cell clusters. The primary reference dataset for B cell annotation was GSE193868, a cellular indexing of transcriptomes and epitopes sequencing (CITE-seq) dataset with well-characterized MZB clusters, MZB-1 and MZB-2 [15]. In addition, two other databases, HumanPrimaryCellAtlasData (HPCA) and BlueprintEncodeData, were used as secondary and tertiary references. SingleR was employed to check if the predicted annotations for a query cluster were consistent across different reference datasets. If the predictions were not reliable, we evaluated whether well-known marker genes of B cell types were among the top differentially expressed genes (DEGs) in the query clusters, and assigned the most likely identity to each cell cluster accordingly [25,26]. Finally, signatures involved in MZB and GCB development, as indicated by the Molecular Signatures Database (MSigDB) C5 database, were used to calculate kernel density and key gene scores, confirming the validity of our B cell subcluster annotations.

DEGs were deemed significant using the Wilcoxon rank sum test with a log fold change threshold of 0.25 and expressed in 10 % of either population (*Seurat*::*FindMarkers* function). The positive markers of B cell subclusters are summarized in **Table S13**.

### scRNA-seq downstream analysis

The downstream analysis comprised Gene Ontology (GO) analysis, Gene Set Variation Analysis (GSVA), Gene Set Enrichment Analysis (GSEA), and cell communication analysis. GO analysis was employed to elucidate the biological significance of gene expression differences in B cell subclusters between HNSCC patients and healthy individuals. GO enrichment of gene clusters was performed using the clusterProfiler package (version 4.4.4) on identified DEGs with an average log2 fold change (log2FC) greater than 0.15 and an adjusted *P* value below 0.05 [27]. This approach allowed for the identification of the most significantly altered genes between the normal and pathological states. The enrichment was defined with a *P* value cutoff of 0.05 in the *clusterProfiler::compareCluster* function.

GSVA, a non-parametric and unsupervised method, was applied to further quantify the pathway and biological process activity across B cell subclusters. This analysis was done using the GSVA package (version 1.46.0) with the default settings [28], and Reactome (https://reactome.org) served as the reference database for identifying relevant pathways.

Cell communication analysis was done using CellChat (version 1.6.1), with the CellChatDB.human database as reference database [29]. CellChatDB.human contains literature-supported human ligand-receptor (L-R) interactions curated from signaling pathway database and recent peer-reviewed experimental studies. For a detailed manifestation of cell–cell interaction, a subcluster annotation was performed in the whole dataset including all the CD45^+^ TILs. The resolution for *FindClusters* function was set as 0.8. The finely annotated labels stored in HPCA database were referred to, as were the cell clusters annotated by the original peer-reviewed research articles from the four scRNA-seq datasets GSE139324, GSE164690, GSE181919, and GSE234933. After annotation of the lymphocyte subpopulations, including T cells, monocytes, dendritic cells (DCs) and macrophages, the B cell subpopulation labels were imported into the active idents, thus building a profile of comprehensive T cells, B cells, monocytes, and DCs subpopulations. The gene lists for individual immune cell subclusters are collectively shown in **table S14**. CellChat was employed to identify and quantify cell–cell communication networks, focusing on signaling pathways and L-R interactions specific to MZB-1 and MZB-2 clusters.

GSEA was conducted to compare the pathway enrichment between MZB-1 and MZB-2 clusters, using GO, Kyoto Encyclopedia of Genes and Genomes (KEGG), and MSigDB C7 (immunologic signature gene sets) as reference databases [30]. Within the MSigDB C7, we specifically selected gene sets related to NOTCH signaling pathways. Using these gene sets, we performed GSEA to analyze the functional difference of MZB-2 in comparison to MZB-1 within primary HNSCC tumor tissues.

### Survival analysis

The Cancer Genome Atlas (TCGA) HNSC dataset was utilized for survival analysis to investigate the prognostic implications of varying tissue proportions of MZB-1, MZB-2 and GCB cells in HNSCC patients. We employed CIBERSORTx, a deconvolution method that uses gene expression data from bulk RNA-seq to estimate the proportions of different cell types within a heterogeneous tissue sample [31]. The gene expression matrix from the integrated scRNA-seq cohort, which included detailed TIL cell type annotation, was used as signature matrix. The TCGA-HNSC dataset served as the query dataset. Through CIBERSORTx, we deconvoluted the TCGA-HNSC bulk RNA-seq data to profile immune cell types in the complex tissue samples of HNSCC patients. The HPV status of patients in the TCGA HNSC cohort was reannotated by oncoviral genotyping [32].

Survival curves were generated using the Kaplan-Meier method, and survival differences were statistically evaluated via the Mantel-Cox log-rank test. Optimal cut-off values for MZB-1 and MZB-2 were determined using the maxstat method to identify precise thresholds for prognostic group stratification.

### Spatial transcriptomics (ST) analysis

We analyzed the spatial distribution of MZB clusters using the publicly available ST dataset GSE220978. As part of the preprocessing, spots with fewer than 200 detected features were excluded. The remaining data were normalized using the *SCTransform* method [33]. This was followed by dimensionality reduction using PCA and unsupervised graph-based clustering performed with Seurat’s *FindNeighbors* and *FindClusters* functions, focusing on the top 30 PCs. Cluster assignments were visualized with UMAP and spatial representations using Seurat’s *SpatialDimPlot* function.

For cell type deconvolution, we selected the scRNA-seq cohort GSE181919 as the reference dataset. This dataset was downloaded, imported into R, normalized using *SCTransform*, dimensionality reduced, and then clustered using the Louvain method. The existing cell type annotations in this dataset served as reference. We employed Seurat's Label Transfer function with its default parameters for reference-based deconvolution [34], to discern tumor cells and TIL populations within Visium spots. Anchors between the reference and query datasets were identified with *FindTransferAnchors*, and cell type labels were transferred using *TransferData*, assigning the dominant cell type to each spatial spot, based on the highest classification probability. These inferred annotations were cross-validated against the pathologist's annotations and multiplex immunofluorescence data presented in the original publication [35]. Finally, for more precise MZB cluster annotation, we utilized our extensively labeled scRNA-seq cohort as the reference and the GSE220978 Visium samples as the query dataset. This analysis was again conducted using the Seurat Label Transfer function, ensuring consistency of the methodology.

### Statistics, data analysis, and visualization

All statistical analyses, data processing, and visualizations were conducted using GraphPad Prism (version 10.0) and R (version 4.0.3 and 4.2.0) [36]. Specialized visualizations such as Circos plots were constructed using the circlize R package [37]. Volcano plots were generated using the EnhancedVolcano package [38], while heatmaps were visualized using the ComplexHeatmap package [39]. Figures related to single-cell sequencing were created using Seurat (version 3.2.2), ggplot2, and ggpubr packages [[40], [41], [42]].

The normality of data distribution was assessed using Shapiro-Wilk’s normality test, while Levene’s test was employed to evaluate homogeneity of variances. Residuals with a normal distribution (*P* > 0.05) and homogeneity of variances (*P* > 0.05) were considered valid. Extreme outliers, defined as points three times the interquartile range, were removed.

For non-normally distributed variables, the Mann-Whitney test was applied to compare two groups. For variables that passed the normality test, unpaired *t*-test with Welch’s correction was used to compare two groups. Multiple group comparisons were performed using one-way ANOVA followed by Bonferroni post-hoc analysis. Data presentation followed the conventions of mean ± standard error of means or as box plots displaying medians, quartiles, and ranges, unless specified otherwise. Boxplots adhered to Tukey's style, where lower and upper hinges corresponded to the 25th and 75th percentiles, and whiskers extended from the hinge to the largest value no further than 1.5 times the interquartile range. Data beyond the whiskers were considered as outlying points and plotted individually. Significance levels were defined as follows: *P < 0.05; **P < 0.01; ***P < 0.001; ****P < 0.0001.

## Results

### Detection and characterization of important B cell subpopulations in HNSCC patients

**Fig. 1:**
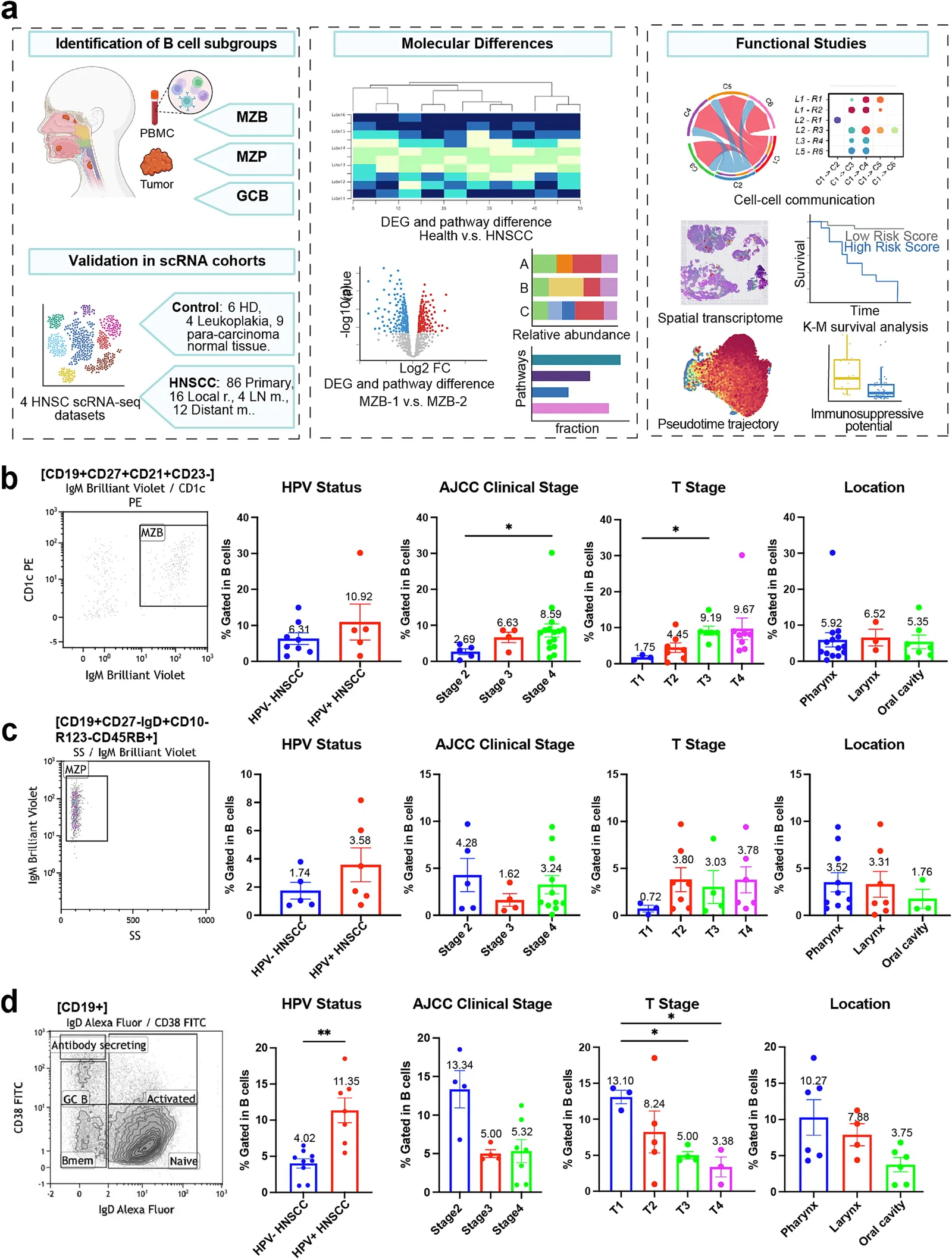
**Flow cytometry analysis of B cell subtypes in HNSCC.** (**a**) Schematic research flow chart. (**b-d**) Representative flow cytometry plots showing the gating strategies for identifying MZB (b), MZP (c), and GCB (d) in dissected HNSCC tumor samples, and distribution of cells by clinical parameters: HPV, AJCC clinical stage, T stage, and tumor location. Each dot represents an individual tissue sample. Cell proportions were tested using unpaired *t*-test with Welch’s correction or one-way ANOVA with Bonferroni post-hoc analysis.

Based on our previous findings in mice, in which we were able to demonstrate an increase in MZB with immunosuppressive potential in tumor tissue during tumor progression, it was first important to determine whether these findings are transferable to the human situation [3]. Therefore, detailed analyses of the B cell compartment with potentially pro- or anti-tumorigenic properties in tumor tissues and the peripheral blood of HNSCC patients were performed (Fig. 1a). To achieve this, three independent panels were developed for the identification of MZB, MZP, and GCB. Detailed gating strategies are illustrated in **Fig. S1a-c**. Flow cytometry results revealed the presence of MZB (Fig. 1b**, Fig. S2a-f**), MZP (Fig. 1c**, Fig. S3a-f**), and GCB (Fig. 1d**, Fig. S4a-f**) in both the TME and the circulation of HNSCC patients, marking a first-of-its-kind discovery of the MZB and MZP populations in squamous epithelial-derived tumors of the head and neck region.

MZB were gated as CD19^+^CD27^+^CD21^+^CD23^-^CD1c^+^IgM^+^, as previously described [15,[43], [44], [45], [46]]. Notably, we observed an enrichment of MZB in human papillomavirus (HPV)-positive (HPV^+^) HNSCC patients as opposed to their HPV-negative (HPV^-^) counterparts, evident in peripheral blood (**Fig. S2c**) as well as in tumor tissue (Fig. 1b). No significant disparity in MZB proportion were observed based on tumor location within our cohort (**Fig. S2d,** Fig. 1b). Specifically, in the circulation, MZB were noted to be more abundant in patients at the T2 stage (**Fig. S2e)**. The frequency of MZB was positively correlated with the progression of tumor stages as classified by the Eighth Edition American Joint Committee on Cancer (AJCC), a trend consistent across both PBMC and tumor tissues (**Fig. S2f,** Fig. 1b).

MZP were identified using the gating strategy CD19^+^CD27^-^IgD^+^CD10^-^R123^-^CD45RB^+^IgM^+^, following previous published studies [44,47]. A modest enrichment of MZPs was observed in HPV^+^ tumors compared to HPV^-^ tumors (Fig. 1c); however, this trend was not reflected in peripheral blood samples (**Fig. S3c**). Within our cohort, the proportion of MZPs did not significantly vary based on tumor location (**Fig. S3d,** Fig. 1c). MZP presentation in blood showed a decreasing trend with advancing AJCC T stages (**Fig. S3e**) and clinical stages (**Fig. S3f**), particularly when adjusted for cell numbers relative to each patient’s white blood count. However, the same trend was not observable in tumor tissues (Fig. 1c).

GCB were gated as CD38^+^IgD^-^ [48]. GCBs were more abundant in the tumor tissues of HPV^+^ patients compared to HPV^−^ individuals (Fig. 1d); whereas this difference was less pronounced in peripheral blood (**Fig. S4c**). In HNSCC tissues, GCB frequency showed a decreasing trend with advancing AJCC clinical stages and T stages (Fig. 1d). The anatomical distribution of GCBs showed a higher proportion in tumors of the pharynx, followed by the larynx and oral cavity, although this trend did not reach statistical significance (Fig. 1d). In contrast, no clear trends in GCB proportions were observed in circulation, regardless of primary tumor location (**Fig. S4d**), AJCC T stage (**Fig. S4e**) or overall clinical stage (**Fig. S4f**).

In addition, we observed distinct differences in other common B cell subsets between HPV^+^ and HPV^-^ HNSCC patients. CD38^hi^ antibody-secreting cells and CD38^-^IgD^-^ memory B cells exhibited a modest increase in HPV^−^ tumors compared to HPV^+^ cases. Notably, we revealed a higher abundance of CD27^-^CD21^lo^ tissue-like memory B cells and CD27^+^CD21^+/-^ resting/activated memory B cells in HPV^+^ tumors relative to their HPV^−^ counterparts, suggesting a more diverse or activated memory B cell landscape in the HPV^+^ tumor microenvironment **(Fig. S5)**.

In summary, we identified MZB, GCB, and MZP in both the TME and the circulation. Our findings indicate a tendency of decreased GCB and increased MZB in tumor tissues with higher AJCC pathological stages, particularly the T stage. GCB were more abundant in individuals with earlier T stages, whereas MZB increased with advancing T stage within the TME. Additionally, more MZBs and GCBs were identified in HPV^+^ tumors in comparison to HPV^-^ HNSCC.

### Dissecting TIB complexity using single-cell transcriptomics reveals distinct functional differences

To corroborate our findings, we conducted scRNA-seq analysis using four publicly available datasets encompassing a total of 118 patients with HNSCC, along with control samples from healthy individuals for comparative analysis [[18], [19], [20], [21]]. Focusing exclusively on CD45^+^ cells from four independent scRNA-seq datasets, rigorous quality control, normalization, and integration were performed. Overall, 288,638 CD45^+^ cells were within the integrated dataset. The integrated data from various tissue origins and donors exhibited a high level of heterogeneity of cells in each cluster, as illustrated in the UMAP plots (**Fig. S6a-c**).

**Fig. 2:**
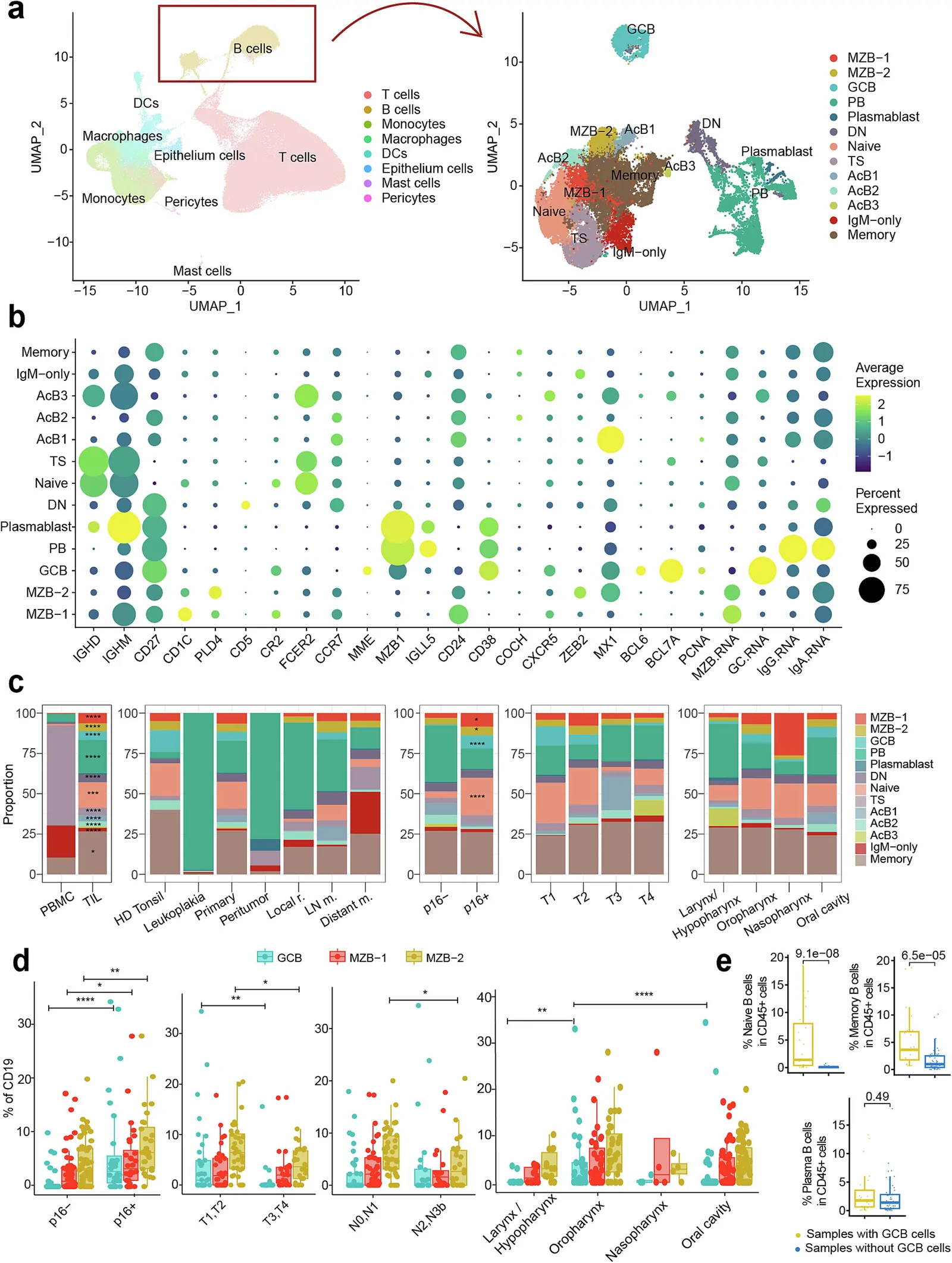
**ScRNA-seq analysis of CD45^+^ TILs and B cell subtypes.** (**a**) UMAP visualization of all CD45^+^ TILs and B cell compositions colored by cell clusters, annotated on the basis of Seurat unsupervised clusters. (**b**) Dot plot illustrating marker gene expression for B cell subtypes. The following gene groups were used: MZB.RNA (*CD1C, PLD4, CDH17, DOCK11, PTK2B, DLL1, LFNG, MFNG, NOTCH2, DOCK10,* and *BCL3*); GC.RNA (*MME, PCNA, BCL6*, and *BCL7A*); IgG.RNA (*IGHG1, IGHG2, IGHG3, IGHG4*); IgA.RNA (*IGHA1* and *IGHA2*). (**c**) Stacked bar plots showing the proportions of B cell subsets in HNSCC patients from different sources (PBMCs or tissue-resident TILs) and in tissues under various pathophysiological conditions, including disease progression, HPV status, AJCC T stages, and tumor locations. (**d**) The comparisons of GCB, MZB-1 and MZB-2 proportions in primary HNSCC tissues stratified by HPV state, tumor T stage, N stage, and tumor localization. (**e**) Boxplots comparing the abundances of naive B cells, memory B cells, and PBs between tumors with and without GCB cells. Only primary HNSCC tumor samples are shown. r., recurrence; m., metastasis. Each dot represents an individual tissue sample. Cell proportions were tested using two-sided unpaired *t*-test with Welch’s correction or one-way ANOVA with Bonferroni post-hoc analysis.

For the initiatory annotation, major immunogenetic clusters were delineated, encompassing T cells, B cells, monocytes, macrophages, DCs, epithelial cells, mast cells, and pericytes (Fig. 2a). Subsequently, we specifically isolated the B cell population for a secondary round of subcluster annotation. A reliable approach, utilizing the reference CITE-seq dataset GSE193868, the Molecular Signatures Database (MSigDB), and employing kernel density estimation and priori knowledge on important B cell markers, was used for the identification of B cell subclusters (see **Methods**).

Thirteen distinct B cell subclusters were identified, as depicted in Fig. 2a. The transcript profiles of these B cell subpopulations were summarized in Fig. 2b. Two MZB clusters defined by the presence of *CD27*, *CR2* (*CD21*), *IGHM*, *IGHD*, *CD1C*, and *PLD4* transcripts were discovered.

Three clusters expressing one or few of the activation markers *CD86*, *FCER2*, *MALAT1 and BCL7A*, were designated AcB1 to AcB3 [49,50]. AcB1 exhibited a strong signature of interferon-regulated genes, including *IFI6*, *IFIT1*, and *MX1,* suggesting prior activation through the antiviral mechanism. AcB2 closely resembled GCB, expressing B cell receptor (BCR)-regulated genes *JUN* and *FOS* [51]*.* AcB3 included several genes from the heat shock protein family, associated with carcinoma and infectious diseases [52,53]. One non-adjacent cluster lacking both CD27 and IgD was termed double negative (DN) B cells (Fig. 2b).

The relative abundance of B cell subsets was compared across HNSCC patients with different sample sources (PBMCs or tissue-resident TILs) and tissues under various pathophysiological conditions (Fig. 2c). The composition of B cell subsets differed significantly between tumor sites and circulation. Both MZB and GCB subsets were consistently detected in peripheral blood and tumor tissues, but their relative abundance was higher in tumor tissues compared to circulation. AcBs, particularly AcB3, were predominantly found in primary HNSCC tumors and were largely absent from circulation and HD tonsil tissues. MZB clusters were identified in HD tonsil samples, primary HNSCC tumors, and in cases of local recurrence, LN metastasis, and distant metastasis. However, neither MZB cluster was detected in leukoplakia or peritumor normal tissues. GCB cells were mainly observed in healthy tonsil, primary HNSCC tissue, and LN metastasis of HNSCC. When comparing tumor samples based on HPV infection status, the proportions of MZB-1, MZB-2, GCB, and naive B cells were significantly higher in HPV^+^ tumors compared to HPV^-^ samples. These observations were consistent with flow cytometry data.

Examining the proportion of TIBs in primary HNSCC tumors revealed some interesting patterns. Flow cytometry data indicated an increase in MZB populations with advancing tumor T stages. In contrast, scRNA-seq analysis showed that both MZB and GCB cells were more prevalent in early T stage HNSCC (T1 and T2) but declined in later T stages (T3 and T4). Additionally, the proportion of the MZB-2 cluster decreased as the tumor involved more LNs (N stage). Notably, a higher abundance of GCB was observed in tumors located in the oropharynx. However, no significant differences were found for the MZB-1 and MZB-2 clusters concerning tumor location (Fig. 2d). The abundances of naive and memory B cells appeared to be significantly higher in tumors with GCB cells than those without, whereas no significant difference was observed in the prevalence of plasma B cells (PBs) (Fig. 2e).

### B cell heterogeneity in HNSCC patients and healthy individuals highlights metabolic and viral pathway alterations

**Fig. 3:**
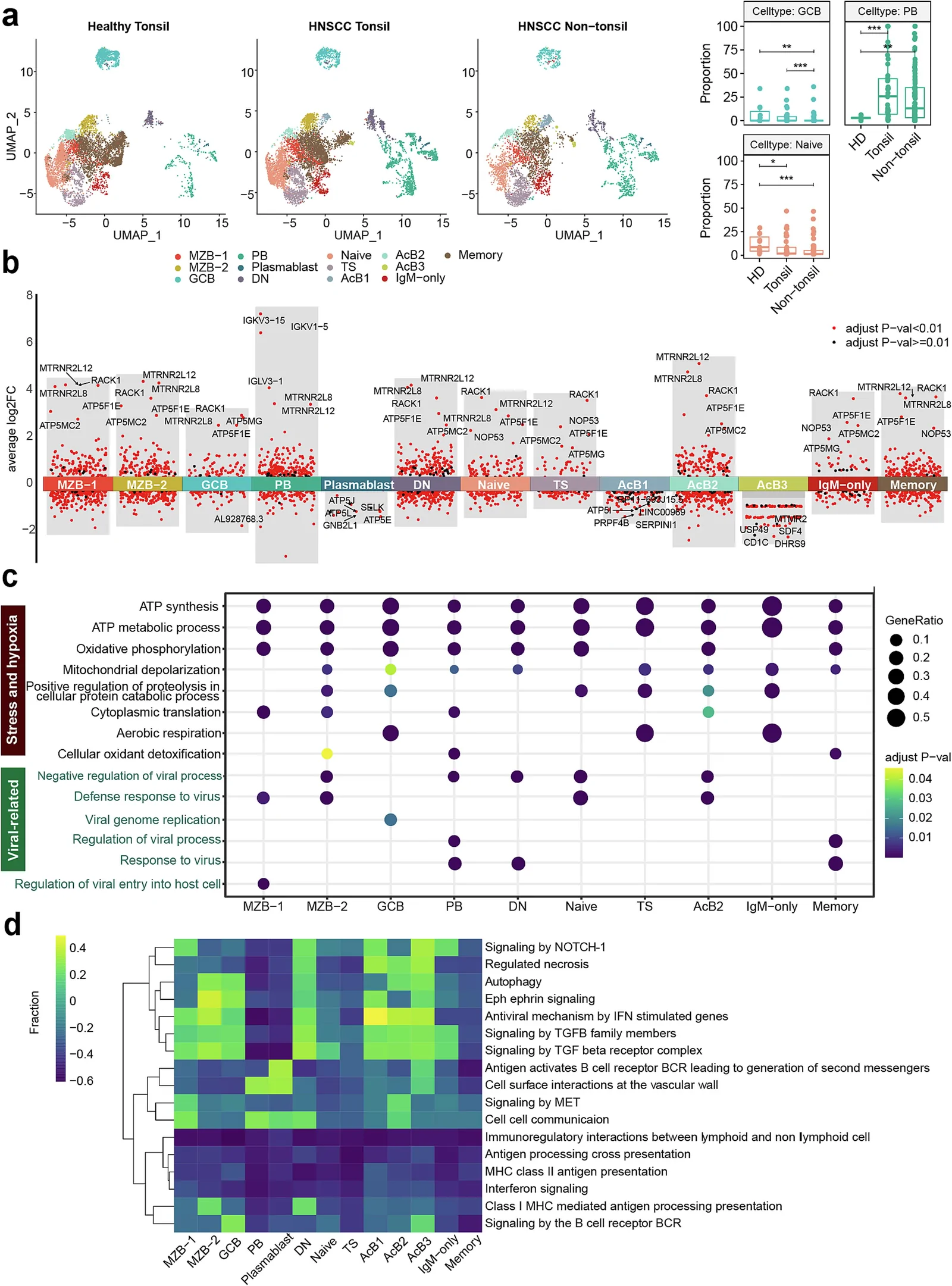
**Comparative analysis of MZB in HNSCC patients and healthy donors.** (**a**) Proportion of B cell subpopulations across healthy tonsils (non-tumor), primary tonsillar HNSCC, and primary non-tonsillar HNSCC samples. B cell proportions were statistically compared using one-way ANOVA. Significant differences among cell clusters were further examined with Bonferroni post-hoc tests. (**b**) Differential gene expression highlighting top five DEGs across thirteen B cell subclusters. Genes with an adjusted *P* value < 0.01 are marked in red, while those with adjusted *P* values between 0.01 and 0.05 are shown in black. (**c**) GO analysis for biological process among B cell, illustrating differences between HNSCC patients and healthy individuals. (**d**) GSVA analysis of enriched pathways within each B cell subcluster. The pathways displayed are selected from the top 20 enriched pathways according to the Reactome database. Enrichment scores were compared among clusters using standardized fractions.

To discern potential disparities in B cell populations expressed in HDs versus HNSCC patients, we compared the relative abundance and transcriptomic profiles of B cell clusters between HD tonsils and primary HNSCC. Regardless of anatomical tumor origin (tonsillar or non-tonsillar), HNSCC tissue harbored a higher frequency of PBs compared to HDs. In contrast, GCB and naive B cells were more prominent in HDs than in HNSCC tissues **(**Fig. 3a**)**.

Transcriptomic comparison of B cell subclusters revealed alterations in gene expression between HNSCC and HDs. Among the top DEGs (adjusted *P* value < 0.01), several were implicated in mitochondrial ATP synthesis, including *ATP5MC2*, *ATP5F1E*, *ATP5MG*, *ATP5E*, *ATP5L*, and *ATP5J*. Elevated expression of apoptosis-associated genes (*MTRNR2L8* and *MTRNR2L12*) and immunoglobulin gene segments (*IGKV1-5*, *IGKV3-15*, *IGLV3-1*) were also observed, reflecting enhanced immunoglobulin rearrangement and stress responses (Fig. 3b).

GO Biological Process (BP) enrichment analysis further identified key pathways elevated in HNSCC-associated B cells, particularly those involved in mitochondrial metabolism (e.g., oxidative phosphorylation, ATP biosynthesis, aerobic respiration), hypoxia responses, and oxidative stress detoxification (Fig. 3c). Additionally, pathways associated with antiviral immunity—including viral genome replication, defense response to virus, and viral entry regulation—were enriched in HNSCC patients. These changes may stem from viral etiology, such as HPV-driven oncogenesis.

To further dissect the difference between HPV^+^ and HPV^-^ HNSCC, ReactomePA-based pathway enrichment was performed. HPV^+^ B cell clusters demonstrated elevated enrichment in pathways related to viral mRNA translation, BCR activation leading to second messenger generation, and major histocompatibility complex (MHC)-I and MHC-II-mediated antigen presentation (**Fig. S7**).

In summary, comparative analysis of B cell subsets in HNSCC patients display altered abundance and transcriptomic activity compared to HDs, particularly in antiviral and metabolic pathways. Enrichment of PBs in tumor tissues, coupled with metabolic and immune activation signatures, suggest a tumor-adaptive or virus-induced reprogramming of B cell function.

### MZB-mediated interactions with T cells and APCs reveal enhanced immunomodulatory roles in HNSCC

To evaluate B cell function states in HNSCC patients, we applied GSVA. MZB-1, PBs, and DN B cells exhibited a more prominent role in mediating cell–cell communication. AcB1-3 clusters, along with the MZB-2 cluster, demonstrated a pronounced activation of antiviral responses, particularly through interferon (IFN)-stimulated genes and the Eph/ephrin signaling pathways. These clusters also demonstrated high involvement in transforming growth factor beta (TGF-β)/TGF-β receptor (TGFBR) signaling pathways. Both MZB-2 and DN B cells showed significant upregulation of genes involved in MHC-I mediated antigen processing and presentation. Interestingly, MZB-1 exhibited a marginal increase in NOTCH-1 signaling activity compared to MZB-2, although there was no notable difference in their engagement with the NOTCH-2 pathway (Fig. 3d).

**Fig. 4:**
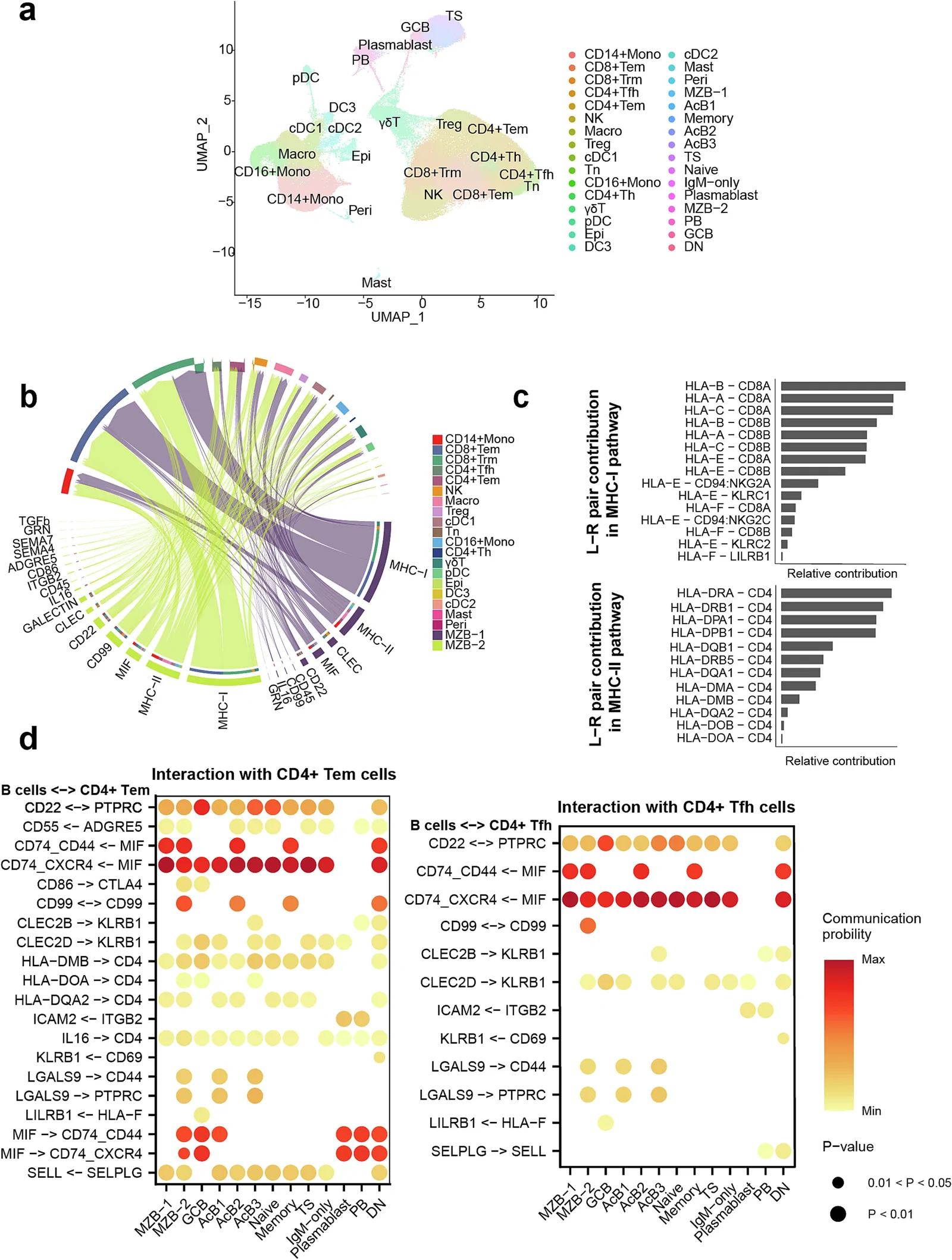
**Functional characterization of MZB-1 and MZB-2 within primary HNSCC tumor tissue.** (**a**) UMAP visualization of CD45^+^ TIL composition in the scRNA-seq cohort colored by cell subsets, annotated on the basis of Seurat unsupervised clusters with main T cell subpopulations: naive T cells (Tn), CD8^+^ tissue-resident memory T cells (Trm), CD4^+^ and CD8^+^ effector memory T cells (Tem), CD4^+^ follicular helper T cells (Tfh), regulatory T cells (Treg), and γδ T cells; four DC populations: conventional DCs (cDC1 and cDC2), plasmacytoid DC (pDC), and DC3; two monocyte populations (CD14^+^ and CD16^+^), and mast cells, macrophages, and NK cells. Two small clusters −- epithelial cells and pericytes −- were also detected. (**b**) Circos plots showing the main cell communication pathways of MZB-1 cluster (purple) and MZB-2 cluster (light green). Interactions are visualized by lines, with the influenced cell type situated in the terminal point. (**c**) Main ligand-receptor pairs in MHC class I and II pathways. (**d**) Significant ligand-receptor pairs between CD4^+^ T cells (CD4^+^ Tem and CD4^+^ Tfh) and each B cell subset within tumors.

Since the GSVA analysis illustrated high association of the MZB-1 cluster with cell–cell communication, we conducted cell–cell communication analysis using CellChat. We annotated our data with detailed subgroups of T cells, DCs, monocytes, and macrophages, defining a total of 32 clusters (Fig. 4a). These included eight T cell subpopulations: naive T cells (Tn), CD8^+^ tissue-resident memory T cells (Trm), CD4^+^ and CD8^+^ effector memory T cells (Tem), CD4^+^ follicular helper T cells (Tfh), regulatory T cells (Treg), and γδ T cells; four DC subpopulations: cDC1, cDC2, pDC, and DC3; and two monocyte types: CD14^+^ and CD16^+^ monocytes. Additionally, we detected clusters of mast cells, macrophages, nature killer (NK) cells, as well as two CD45^-^ cell clusters, epithelial cells and pericytes, which were retained after the CD45^+^ sorting process (Fig. 4a).

A cell communication map revealed that both MZB-1 and MZB-2 clusters predominantly engaged in MHC-I and MHC-II interaction pathways with CD4^+^ and CD8^+^ T cells (Fig. 4b). *HLA-A*, *HLA-B* and *HLA-C* were the most frequent MHC-I ligands to interact with *CD8A* and *CD8B* receptors of CD8^+^ T cells, while the most frequent MHC-II related L-R pairs were the *HLA-DRA*, *HLA-DRB1*, *HLA-DPA1* and *HLA-DPB1* ligands from MZB clusters to interact with the *CD4* receptor (Fig. 4c).

Further analysis showed that, compared to MZB-1, MZB-2 exhibited enhanced interaction potential with CD4^+^ and CD8^+^ T cells, ranking among the highest within TIBs (**Fig. S8a**). While robust MHC-I and MHC-II interaction pathways were observed, none of the L-R pairs identified suggested a classical costimulatory role (**Fig. S8b**). In particular, no CD80/CD86–CD28 interactions were detected between either MZB cluster and CD4^+^ or CD8^+^ T cells. Instead, MZB-2 engaged CD4^+^ Tem and CD4^+^ Tfh cells through multiple immunoregulatory pathways, including the inhibitory *CD74*–*MIF* and *LGALS9*–*CD44/PTPRC* L-R axes, and the *CD99*–*CD99* interaction, which exerts dual effects on T cell adhesion/activation and apoptosis (Fig. 4d).

Beyond T cell interactions, MZB-2 also interacted with professional antigen-presenting cells (APCs) such as CD14^+^ monocytes, cDC1 and macrophages through adhesion-related L-R pairs such as *ADGRE5–CD55* and *ITGB2–ICAM1/2* (Fig. 4b**, Fig. S8b**).

To assess whether these interactions were tumor-specific, we compared MZB signaling between primary HNSCC tumors and HD tonsil tissues. In HDs, both MZB-1 and MZB-2 clusters retained activation of MHC-I and MHC-II pathways (**Fig. S9a**). Notably, however, the MZB-2–CD4^+^ Tem interaction demonstrated divergent L–R usage: the *CD86–CTLA-4* coinhibitory pair was detected exclusively in HNSCC, whereas the *CD86–CD28* costimulatory pair was restricted to HDs (**Fig. S9b**). Pathway intensity analysis further revealed that in HDs, the CD40 costimulatory pathway was significantly enriched, while in HNSCC patients, alongside pronounced MHC-I pathway activation, multiple immunosuppressive pathways—including PD-L1 and IFN-II signaling—were preferentially expressed (**Fig. S9c**).

Overall, our data indicate that tumor-associated MZB clusters predominantly interact with T cells through MHC-I and MHC-II pathways, yet fail to deliver the requisite costimulatory signals necessary for full T cell activation. Compared to MZB-1, MZB-2 exhibits an enhanced propensity to interact with CD4^+^ T cells and APCs, potentially acquiring APC-like functions. Although such cellular interactions were also observed in HDs, indicating they are not tumor-exclusive, HNSCC patients displayed greater reliance on MHC-I–mediated signaling and an enrichment of immunoregulatory interactions, reflected by the upregulation of immunosuppressive L-R pairs and the absence of stimulatory secondary signals, most notably the CD86–CD28 costimulatory axis, which was preserved in HD samples.

### Activated phenotype of MZB-2 reflects advanced maturation and immunomodulatory potential in HNSCC

**Fig. 5:**
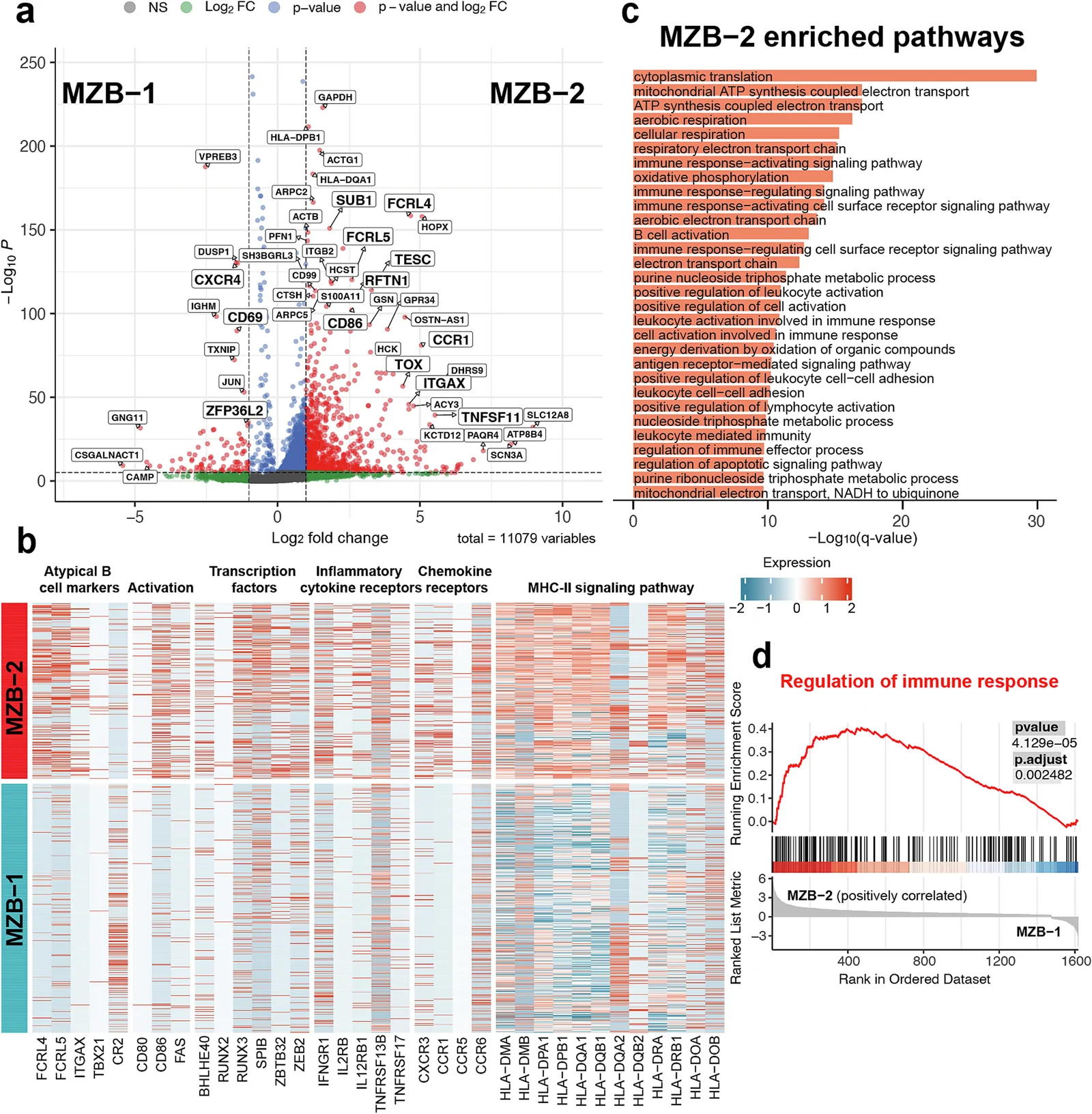
**MZB-1 and MZB-2 difference within primary HNSCC tumor tissue**. (**a**) Volcano plot of differentially expressed genes between MZB-1 and MZB-2 clusters. (**b**) Expression of selected genes differentially expressed between MZB-1 and MZB-2 clusters (BH-adjusted *P* value < 0.01, two-sided unpaired Wilcoxon test). (**c**) Differences in pathway enrichment scored per cell by GSEA comparing MZB-2 to MZB-1 cluster. GO as reference database. (**d**) GSEA Enrichment plot found regulation of immune response to be one of the most significantly enriched pathways in MZB-2 compared to MZB-1, with a nominal *P* value < 0.001. GOBP as reference database.

We next investigated differences between MZB-1 and MZB-2 clusters. As can be noted in the volcano plot (Fig. 5a), genes uniquely expressed in MZB-1 included chemotaxis (*CXCR4*), lymphocyte proliferation and function (*CD69*, *ZFP36L2*). Conversely, MZB-2 expressed genes linked to lymphocyte maturation (*TOX*), myeloid cell differentiation (*TESC*), and B cell functional maturation (*CD86, HLA* alleles, *RFTN1*).

*SUB1* (PC4), which is involved in the negative regulation of DNA metabolic process and the regulation of transcription by RNA polymerase II, was found to be highly expressed in MZB-2. *SUB1* plays a role in genome stability and the stabilization of matured B cells [54,55], as well as in inhibiting virus infection [56,57]. *ITGAX* and *CCR1*, implicated in monocyte adhesion and chemotaxis, were also highly expressed in MZB-2, compared to MZB-1. The expression of *TNFSF11* in MZB-2 further implied a role in apoptosis regulation.

We then examined the expression profiles of key genes of interest between the MZB-1 and MZB-2 clusters. MZB-2 predominantly expressed *FCRL4* and *FCRL5*, two atypical B cell markers recently identified as prominent in tumor-associated atypical B cells (TAABs), and implied functional interactions with CD4^+^ T cells [58]. Additionally, compared to MZB-1, MZB-2 displayed transcriptional features of an activated B-cell state, with elevated expression of activation markers, transcription factors, receptors for inflammatory cytokines and chemokines, and MHC-II signaling pathways (Fig. 5b). These findings underscore the advanced functional maturation stage of MZB-2 compared to MZB-1.

The pathway enrichment between MZB-1 and MZB-2 was compared using GSEA analysis. Compared to MZB-1, MZB-2 was significantly enriched in multiple pathways associated with immune response activation, positive regulation of immune cell activation, immune regulation, and cell–cell adhesion, suggesting an immunoactive phenotype with potential regulatory capacity (Fig. 5c). Among these, regulation of immune response was found to be one of the most significantly enriched pathways in MZB-2 compared to MZB-1, with GOBP as reference database (Fig. 5d).

In the HNSCC patient cohort, we did not find a difference in the involvement of NOTCH signaling pathway between MZB-1 and MZB-2, using MSigDB C7 as reference dataset. Given the critical role of NOTCH2 in MZB lineage development [59], we investigated the expression level of NOTCH-2 among all B cell subsets. Highest transcript levels of NOTCH2 (0.115) were found for IgM-only B cells, whereas expression in MZB-1 and MZB-2 was comparatively low (0.066 and 0.027). Consistent with this, we observed substantial gene set overlap among MZB-1, IgM-only, and TS B cells (**Fig. S10b**). The pseudo-time trajectory analysis suggests a potential developmental link between tumorigenic MZB-1 and MZB-2 (**Fig. S10c**).

Collectively, under tumor conditions MZB-2 showed elevated expression of activation markers and enrichment in pathways related to immune response activation compared with MZB-1. These features position the cluster as an immunoactive population capable of shaping immune cell responses. At the same time, MZB-2 expressed multiple transcription factors and receptors for inflammatory cytokines and chemokines, and was highly enriched in several pathways associated with immune regulation. Such immunoregulatory features suggest functional plasticity, enabling the cluster to fine-tune immune responses and, under conditions of chronic activation, potentially shift toward an immunosuppressive state.

### Spatial proximity of MZB clusters to CD4^+^ T cells in the tumor microenvironment suggests potential interactions

**Fig. 6:**
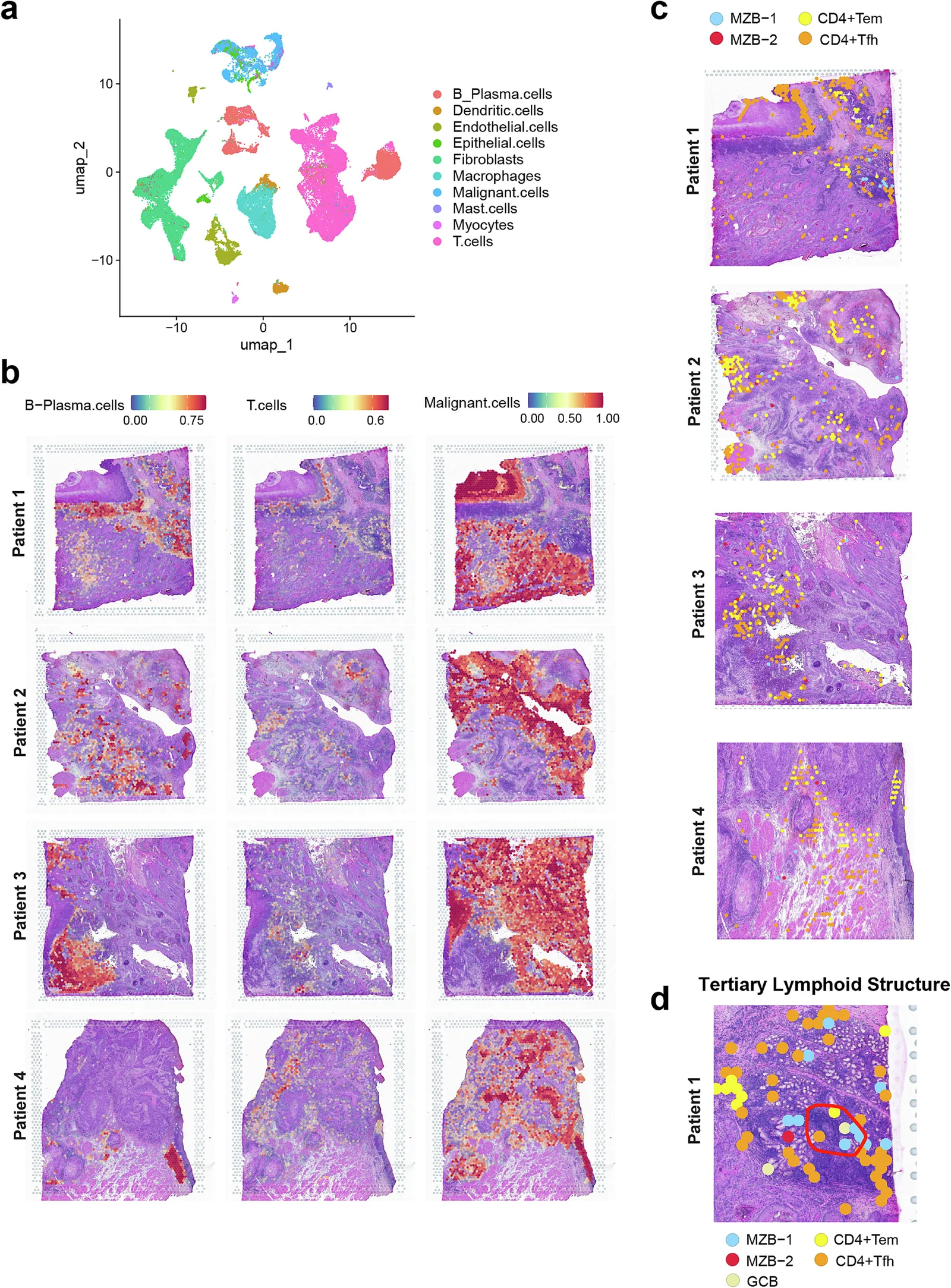
**Spatial transcriptome revealed proximity of MZB and CD4^+^ T cells.** (**a**) UMAP visualization of cell cluster annotations in reference scRNA-seq dataset (GSE181919). (**b**) Spatial deconvolution of GSE220978 Visium demonstrates inferred tumor cells and main TIL distributions. (**c**) Spatial mapping of MZB clusters and CD4^+^ T cell subsets in tumor regions reveals close positional proximity. (**d**) Identification of a TLS (red circle) in Patient 1, showing co-localization of GCB, MZB-1, and CD4^+^ Tfh.

Finally, we analyzed spatial transcriptome (ST) data to elucidate the distribution and localization of MZB clusters in the TME of HNSCC. This approach was applied to a publicly available HNSCC spatial dataset GSE220978, specifically for its comprehensive spatial representation of both tumor and *peri*-tumoral normal tissues from four treatment-naïve HNSCC patients [35]. As a reference, we employed the scRNA-seq dataset GSE181919, which delineates nine major cellular populations—including malignant cells, T cells, B/plasma cells, dendritic cells, and others, as visualized in the UMAP plot (Fig. 6a). Deconvolution of the ST data revealed that the distribution of tumor cells and major TIL populations was highly consistent with histopathological annotations and multiplex immunofluorescence findings reported in the original study (Fig. 6b) [35].

In subsequent analyses, we refined cell-type annotations by using the scRNA-seq dataset as a reference and the GSE220978 Visium data as the query, with a particular focus on MZB and T cell subsets. In regions predicted to be enriched for MZB clusters, we observed a notable spatial proximity to CD4^+^ T cells, particularly CD4^+^ Tfh and CD4^+^ Tem cells (Fig. 6c). These findings suggest a potential functional interaction between MZB clusters and CD4^+^ T cell subsets within the TME.

Notably, in Patient1, we identified a tertiary lymphoid structure (TLS) characterized by the co-localization of GCB, MZB-1 cells, and CD4^+^ Tfh cells (Fig. 6d). This spatial configuration supports the presence of an MZB–Tfh–GC axis, highlighting its potential antitumorigenic characteristics.

### Immune-regulatory potential and prognostic significance of MZB subsets in HNSCC

Given previous studies that MZB populations exhibit regulatory B cell (Breg) phenotypes, marked by the gene expression of *NR4A1-3*, *CD83*, *ENTPD1* (CD39), and *NT5E* (CD73), we explored whether the tumor-associated MZB-1 and MZB-2 subsets also possessed these immunosuppressive markers.

**Fig. 7:**
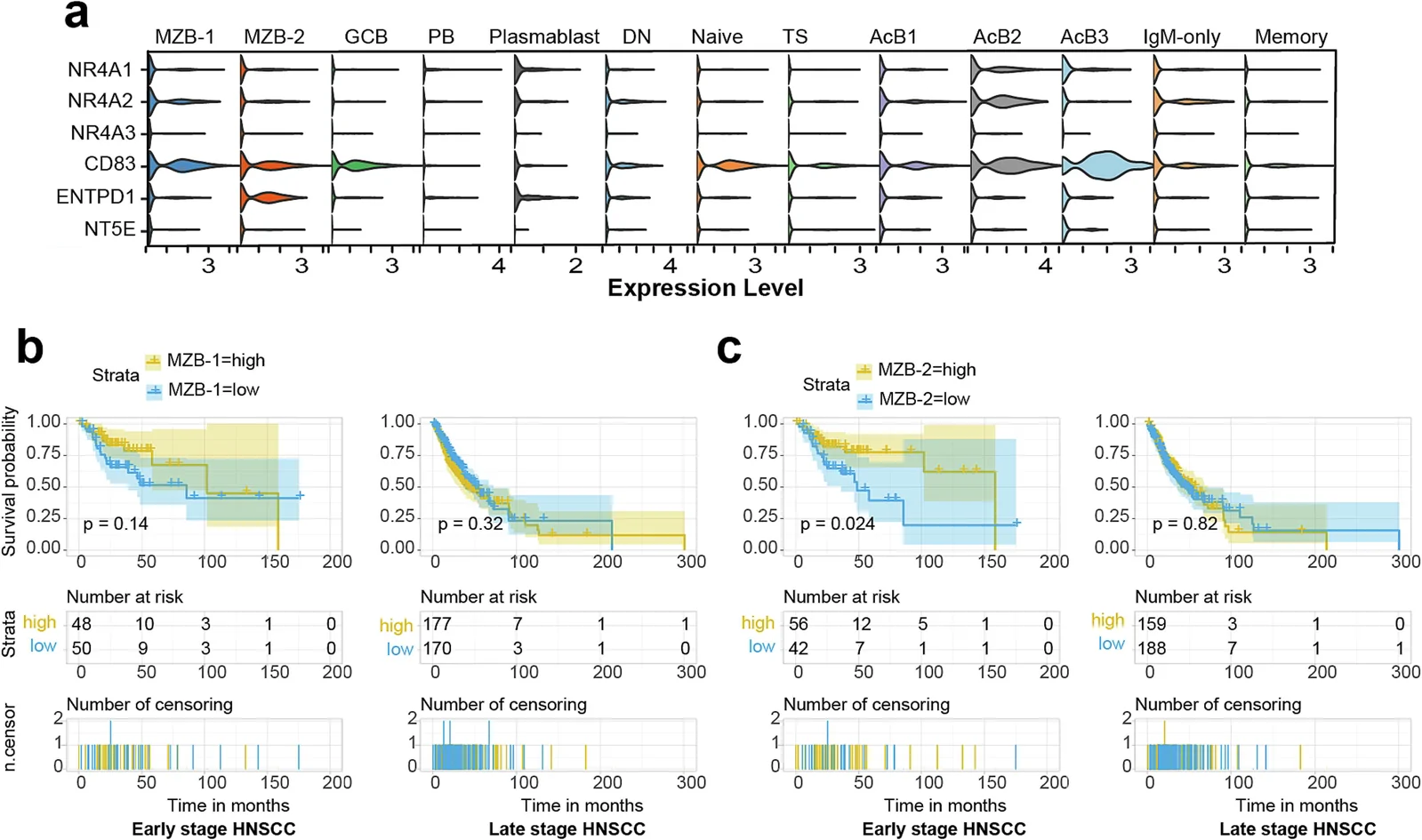
**Immunosuppresive potential of MZB in HNSCC.** (**a**) Individual expression level of important genes acquired from the reference MZB bulk-RNA seq dataset (9), compared among each B cell subsets. (**b**) Survival analysis of MZB-1, MZB-2, and GCB with TCGA HNSC bulk-RNA seq data, stratified by AJCC clinical stages.

At the transcription level, *NR4A1-3* and *CD83* were broadly expressed across nearly all B cell subsets, particularly in the activated B cell (AcB) clusters AcB1-3. This widespread expression makes it difficult to attribute the immunosuppressive phenotype exclusively to MZB, as other B cell subsets also exhibit these immunosuppressive molecules. *ENTPD1* (CD39) and *NT5E* (CD73) were more abundant in the MZB-2 cluster compared to the MZB-1 cluster, but they were also present in other effector B cell populations, including the AcB1-3 clusters (Fig. 7a). Attempts to define discrete Breg clusters within the scRNA-seq dataset did not yield distinct populations.

With the TCGA-HNSC dataset, we constructed an immune infiltration matrix to analyze the proportions and prognostic significance of MZB-1, MZB-2, and GCB cells in HNSCC patients. CIBERSORTx was used to estimate the proportions of different cell types within heterogeneous tissue samples based on bulk RNA-seq data, and the gene expression matrix from our integrated scRNA-seq cohort served as the reference signature matrix. Our analysis revealed a notable increase in MZB-2 proportions within HPV^+^ tumors relative to HPV^-^ counterparts (**Fig. S10a**). Further stratification of survival data using the Kaplan-Meier method demonstrated no significant differences between high and low MZB-1 expression groups in both early and late stage HNSCC. This suggests that MZB-1 may not be a significant independent prognostic factor for survival outcomes in HNSCC (Fig. 7b). However, higher counts of MZB-2 in early-stage HNSCC correlated significantly with improved clinical prognosis (Fig. 7c). Optimal cut-off values for MZB-1 and MZB-2 were determined using the maxstat method, which identify the best thresholds for prognostic stratification (MZB1 lower cutpoint = 1.474, upper cutpoint = 1.854; MZB2 lower cutpoint = 0.469, upper cutpoint = 2.298).

Our findings suggest that MZB-2 exhibits unique immunomodulatory properties — potentially balancing antigen presentation, cytokine signaling, and immune suppression. The favorable prognostic impact of MZB-2 in early-stage HNSCC highlights its potential beneficial role for modulating immune responses in early tumor progression.

## Discussion

For the first time, our study revealed the existence of MZB in the blood and as TILs isolated from HNSCC patients. Given the suggested pro- and anti-immunogenic characteristics of MZB [7], we aimed to uncover their role within the TME in the context of HNSCC development, maintenance, progression, regression, and prognosis. Recently, two MZB subpopulations, MZB-1 and MZB-2, have been identified through mass cytometry and scRNA-seq analysis of various immune-related tissues of HD [15]. Utilizing scRNA-seq and spatial transcriptome, we confirmed the presence of two MZB populations in the TME of HNSCC patients. Based on their distinct marker expression and pathway enrichment, we designated the subset with stronger immunoactive features as MZB-2. Despite several differences from their counterparts in HDs, tumor-associated MZB subsets retained functional similarities, most notably their antigen-presenting capacity.

### The relationship of MZB, MZP and GCB with clinicopathological data

In our study, we examined the proportions of MZB subsets (MZB-1 and MZB-2) and GCB across HNSCC patients with different HPV infection statuses. Both flow cytometry and scRNA-seq data consistently showed higher proportions in HPV^+^ patient samples compared to their HPV^-^ counterparts. This increase in GCB among HPV^+^ patients aligns with findings by Ruffin et al., who noted an enhanced presence of GCB and TLS in HPV^+^ tumors compared to HPV^-^ HNSCC [48]. These observations are consistent with previous findings, showing a “hot” TIME in HPV^+^ HNSCC, featuring more immune infiltration, higher T-cell activation, and greater immunoregulation compared to the “cold” TIME in HPV^−^ cases [60].

Interestingly, discrepancies were noted between the two techniques used for cellular analysis. While flow cytometry indicated an increase in MZB populations correlating with advancing T stage of tumors, scRNA-seq analysis suggested that both MZB and GCB were more prevalent in the early T stages (T1 and T2) of HNSCC but diminished in later stages (T3 and T4). This divergence could be due to differences in unpaired sampling, missing data unbalance and sensitivity between the two methodologies. The inconsistencies between transcriptome and surface protein expression also added to the complexity of interpreting the data.

Additionally, MZB-2, which correlated with a favorable prognosis in early-stage HNSCC, showed a declined frequency and prognostic value in advanced, immunologically 'cold' tumors. This trend suggests that MZB may serve as effective prognostic indicators during the initial phase of tumor development but lose this capability as the TIME evolves. The decline in the proportion of MZB-2 with increased LN involvement (N stage) further underscores the dynamic role of these cells within the evolving tumor landscape.

### Proportion, genomic, and functional differences of B cell subsets between HNSCC and healthy volunteers

Across all primary tumor sites, HNSCC patients exhibited a marked expansion of PBs within tumor tissues compared with PB frequencies in HD tonsils, consistent with antigen-driven activation. This was supported by the upregulation of immunoglobulin V(D)J genes (*IGKV1-5*, *IGKV3-15*, *IGLV3-1*) in tumor-associated PBs. In contrast, GCBs and naïve B cells were enriched in HD tonsils but underrepresented in HNSCC tumors, reflecting their differentiation into effector B cell subsets within the tumor milieu.

Comparative profiling of B cell marker genes between HNSCC and HD tonsils revealed that intratumoral B cells were enriched for metabolic programs, including ATP synthesis, oxidative phosphorylation and mitochondrial depolarization. These pathways are hallmarks of cellular stress and hypoxia, consistent with metabolic adaptations observed across diverse cancers [61,62]. In addition, viral-response pathways were significantly upregulated in HNSCC compared with HD tonsils, reflecting the etiological contribution of HPV^+^ oropharyngeal carcinoma. Indeed, ReactomePA enrichment analysis distinguished HPV^+^ from HPV^−^ tumors, highlighting distinct molecular pathways related to viral activity in several B cell populations derived from HPV^+^ HNSCC compared with their HPV^-^ counterparts.

At the level of cell–cell communication, we observed an enrichment of immunosuppressive signaling pathways, including PD-L1 and IFN-γ, in HNSCC tumors compared with HD tonsils, highlighting a tumor-adaptive shift of B cells toward regulatory functions. A particularly notable alteration was detected in MZB-2–CD4^+^ Tem interactions: in HDs, these were predominantly mediated by the CD86–CD28 costimulatory axis, whereas in HNSCC they were redirected toward the CD86–CTLA-4 coinhibitory axis, reflecting tumor-specific reprogramming of B cell–T cell crosstalk.

### Distinctive features and functional divergence of MZB-1 and MZB-2

The DEGs observed between MZB-1 and MZB-2 clusters reflect distinct functional roles in immune surveillance and response. MZB-1 appears to adopt a more quiescent state, whereas MZB-2 appears to be dynamically involved in advanced stages of the immune response, notably in antigen presentation and immunoregulation.

In terms of developmental origins, in the study by Siu et al. in HDs, distinct differentiation routes for MZB-1 and MZB-2 were presented—namely, an IgM-driven trajectory for MZB-1 and a NOTCH-2–dependent pathway for MZB-2 [15]. While our analyses in HNSCC revealed substantial gene set overlap among MZB-1, IgM-only, and TS B cells, we detected no significant enrichment of NOTCH-2 expression or pathway activity in MZB-2. Instead, pseudo-time trajectory analysis indicated a potential developmental relationship between MZB-1 and MZB-2. Based on the above-mentioned DEGs, pathways, and trajectory analyses, our data suggest that both clusters represent differentiated MZB populations, with MZB-2 exhibiting a more immunoactive and transcriptionally mature phenotype. Further studies involving HNSCC-bearing mice at different time points and functional investigations of MZB-1 and MZB-2 would be necessary to substantiate this model.

The gene sets of MZB-1 and MZB-2 clusters for HNSCC patients overlapped with those reported in HDs [15], with a few notable exceptions. For example, in HDs, DDX21, an RNA helicase, was detected as a positive expressed gene differentiating MZB-2 to MZB-1. DDX21 operates by binding to PB1 and inhibiting RNA virus polymerase assembly [63,64]. In contrast, our findings identified *SUB1*, rather than *DDX21*, as significantly upregulated in the tumorigenic MZB-2 cluster. SUB1 is implicated in the negative regulation of DNA metabolic processes and transcription by RNA polymerase II, playing roles in genome stability, mature B cell stabilization, and acting as a negative regulator of viral infections [[54], [55], [56]]. These differences may indicate context-specific adaptations of MZBs in response to the viral etiology of HNSCC.

Notably, MZB-2 was the only B cell subcluster exhibiting high *FCRL4* expression in the analyzed cohort. This suggests that MZB-2 likely corresponds to the FCRL4^+^ TAABs identified in recent studies, which reported close functional interactions with CD4^+^ T cells and a favorable prognostic association in several cancer types—although in HNSCC this effect was marginal [58]. In our analysis, MZB-2 showed prognostic relevance exclusively in early-stage HNSCC patients and notably lacked the CD80–CD28 costimulatory axis. Collectively, these findings support the hypothesis that in HNSCC, sustained activation of FCRL4^+^ TAABs may lead to the accumulation of exhaustion, which potentially inhibit or fail to support effective anti-tumor immune responses [65,66].

### Antigen-presentation potential of MZB in the tumor microenvironment

Our cell–cell communication analysis revealed that both MZB-1 and MZB-2 clusters predominantly engaged in MHC-I and MHC-II interactions with CD4^+^ and CD8^+^ T cells. Beyond T cell crosstalk, MZB-2 also interacted extensively with professional APCs, including CD14^+^ monocytes, cDC1s, and macrophages, through adhesion- and signaling-related ligand–receptor pairs such as ADGRE5 – CD55 [67] and ITGB2 – ICAM1/2 [68,69]. These interactions suggest that tumor-resident MZB-2 may acquire DC-like properties within the TIME, consistent with the functional plasticity of MZBs in physiological contexts, where MZBs trogocytose the complement component (C) 3 −modified pMHC II complexes from DCs via the complement receptor (CR) 2, and consequently present pMHC II complex to CD4^+^ T cells [45].

In the tumor context, our study provides the first evidence that MZB-2 subsets possess a strong capacity to interact with both CD4^+^ T cells and cDCs via MHC-II pathways. This cooperative antigen presentation may allow MZB-2 to modulate CD4^+^ T cell function within the TIME, thereby extending their known role in immune surveillance to tumor immunology.

The interaction of MZB clusters with CD8^+^ T cells via MHC-I pathways likely reflects cross-presentation, a process in which exogenous antigens, rather than presented conventionally on the MHC-II pathway, are processed and presented on MHC-I molecules by APCs. This mechanism is particularly relevant in non-hematopoietic tumors or viral infections that bypass professional APCs, as it enables CD8^+^ T cell priming against tumor-associated antigens released from dying tumor cells or against extracellular viral antigens [70,71]. In our study, we observed a marked increase in MHC-I pathway activity within the HNSCC tumor milieu compared with HD tonsils, implying a significant role it plays in the tumor immunity. However, it remains controversial whether heightened MHC-I activity within tumors uniformly supports antitumor responses, or whether it may, in certain contexts, be co-opted by tumor cells to evade immune surveillance and blunt the efficacy of immunotherapy [71].

### Immunosuppressive potential of MZB in the tumor microenvironment

Although robust MHC-I and MHC-II pathways were observed, none of the identified L-R pairs suggested a classical costimulatory role. In particular, CD80/CD86–CD28 interactions were absent between either MZB cluster and CD4^+^ or CD8^+^ T cells. However, Further immunoregulatory L-R interactions, such as CD74–MIF [72], CD99–CD99 [73,74], and LGALS9–CD44/PTPRC [75] consolidate the view that MZB-2 plays a immunomodulating role with CD4^+^ Tem and Tfh cells.

The CD99–CD99 homophilic interaction mediates immune cell adhesion and transendothelial migration, and in T cells it promotes proliferation while simultaneously inducing IL-6 and tumor necrosis factor (TNF)-α production and, under certain conditions, apoptosis [73,74]. LGALS9 encodes galectin-9, an S-type lectin implicated in cell adhesion, immune evasion, angiogenesis, and tumor metastasis [76], with multiple members of the galectin family linked to cancer progression and prognosis [[77], [78], [79], [80]]. Functionally, CD74–MIF and LGALS9–CD44/PTPRC constitute well-characterized immunoinhibitory pathways that dampen T cell activation, foster exhaustion, and reinforce an immunosuppressive microenvironment. By contrast, CD99–CD99 displays a context-dependent dual role, enhancing T cell adhesion and activation while also exerting pro-apoptotic effects.

MZB-2 also interacted with CD4^+^ Tem, Treg, and γδ T cells with the CD86–CTLA-4 interaction. CTLA-4, expressed on T cells, serves as a critical negative regulator of immune responses. CTLA-4-expressing Treg cells reduce the expression of CD80/CD86 molecules on APCs via CTLA-4-dependent trogocytosis [81]. This reduction imparts a dual suppressive effect on T cell responses by limiting CD80/CD86 co-stimulation to naïve T cells and enhancing the availability of free PD-L1 for inhibiting PD-1-expressing effector T cells.

Our subsequent attempts to cluster Bregs in the scRNA-seq cohort did not yield distinct Breg clusters, which is consistent with findings in breast cancer and pan-cancer scRNA-seq research [58,82,83]. Our findings, in line with current understanding, suggest that Bregs may represent a transitional phase in B cell development rather than a distinct lineage [84].

## Conclusion

This study provides the first evidence of MZB in both tumor tissue and peripheral blood of HNSCC patients, offering new insights into their role within the disease context. We found that their abundance varied with HPV status and showed distinct interaction patterns with other immune cells, particularly CD4^+^ T cells and cDCs, highlighting the potential of MZB in antigen presentation and immunoregulation.

Among the two MZB clusters that we identified, MZB-2 emerged as the more immunoactive phenotype, characterized by advanced maturation markers and broad participation in immune response pathways. Importantly, MZB-2 displayed a dual immunoregulatory potential. In HDs, it exhibited antigen presentation capacity in conjunction with CD80–CD28 co-stimulation, supporting effective T cell activation. By contrast, in HNSCC patients, MZB-2 engaged CD4^+^ T cells through both MHC-II and CD80–CTLA-4 interactions, suggesting a role in modulating, rather than sustaining, T cell responses. Transcriptomic profiling of MZB-2 revealed its elevated transcription factors, cytokine and chemokine receptors, and enrichment of immune regulatory pathways, which underscores its functional plasticity and implies a potential shift toward immunosuppression state under certain conditions. Survival analysis further revealed that MZB-2 was prognostically favorable in early-stage HNSCC, whereas its impact was negligible in advanced disease.

What limits in our study is the lack of protein-level validation and functional experiments confirming the cellular interactions between MZB and T cells. Currently, we are developing genetically engineered mouse models for the specific depletion of MZB to facilitate in vivo studies. Future studies are designed to conduct proteomic analysis of neoantigens bound to MHC-I from isolated MZB. An additional challenge was our inability to differentiate MZP based on scRNA-seq data, attributable to the absence of a published transcriptomic profile correlating with our phenotype of interest. To paint a more comprehensive picture, further studies incorporating CITE-seq and BCR profiling are necessary to elucidate MZP expression and lineage development within the tumor milieu.

The potential contribution of B cell–mediated antigen presentation to cancer immunotherapy efficacy remains an important but understudied area. Our findings position MZB-2 as both a prognostic biomarker in early-stage HNSCC and a potential modulator of immune responses within the TME. Future prospective studies, particularly in immune checkpoint blockade cohorts, will be essential for us to understand the extent to which MZBs influence patients’ prognosis and immunotherapy outcomes.

## Study approval

Human tumor tissue was obtained during surgery from HNSCC patients with research ethics committee approval (REC; reference number: 90/15) and written informed consent from the patient. Blood samples were obtained from patients with HNSCC and healthy volunteers (non-tumor patients) with written informed consent and REC approval (reference number: 90/15).

## Data availability

The scRNA-seq datasets GSE139324, GSE164690, GSE181919, GSE234933 and the ST dataset GSE220978 used within this study can be found in the GEO database (https://www.ncbi.nlm.nih.gov/geo/). TCGA HNSC data was acquired from the TCGA database (https://portal.gdc.cancer.gov/). The codes can be found in Github (https://github.com/immMLab/Marginal-Zone-B-cells-in-HNSCC).

## Declaration of competing interest

The authors declare the following financial interests/personal relationships which may be considered as potential competing interests: The publication was developed under employment of Ulm University Hospital, and Jiantong Bao is presently employed with Boehringer Ingelheim. The authors declare that the research was conducted in the absence of any commercial or financial relationships that could be construed as a potential conflict of interest.
