# Supplementary material for: Identification of marginal zone B cells in head and neck cancer with immunomodulatory characteristics
Source: J Adv Res. 2025 Nov 29;87:573–90. doi: 10.1016/j.jare.2025.11.070 (PMC13539160; doi:10.1016/j.jare.2025.11.070)
Supplement: Supplementary file 1 [file mmc1.docx]

Supplementary Material

Identification of Marginal Zone B Cells in Head and Neck Cancer with Immunomodulatory Characteristics

Jiantong Bao^1,2^, Jochen Hess^3^, Annika C. Betzler^1^, Zhen Zhang^1^, Miltiadis Tsesmelis^4^, Sebastian Schmid^5^, Gaurav Berry^1^, Patrick J. Schuler^1,3^, Jens Greve^1^, Simon Laban^1^, Thomas K. Hoffmann^1^, Medhanie Mulaw^4*†^, Cornelia Brunner^1,6*†^

^1^Department of Otorhinolaryngology and Head & Neck Surgery, University Medical Center Ulm, Head & Neck Cancer Center of the Comprehensive Cancer Center Ulm; Ulm, Germany.

^2^School of Medicine, Southeast University; Nanjing, China.

^3^Department of Otorhinolaryngology, Head and Neck Surgery, Heidelberg University Hospital; Heidelberg, Germany.

^4^Unit for Single-Cell Genomics, Medical Faculty, Ulm University; Ulm, Germany.

^5^Department of Anesthesiology and Intensive Care Medicine, Ulm University Medical Center; Ulm, Germany.

^6^Core Facility Immune Monitoring, Medical Faculty, Ulm University; Ulm, Germany.

^†^ equal contribution

***** Correspondence:

Cornelia Brunner
[cornelia.brunner@uniklinik-ulm.de](mailto:cornelia.brunner@uniklinik-ulm.de)

Medhanie Mulaw

[medhanie.mulaw@uni-ulm.de](mailto:medhanie.mulaw@uni-ulm.de)

Keywords: marginal zone B cells; head and neck squamous cell carcinoma; antigen presentation; MZB-Tfh-GCB axis.

# 1 Key Resources Table

| **REAGENT or RESOURCE** | **SOURCE** | **IDENTIFIER** | |
| --- | --- | --- | --- |
| **Antibodies** | | | |
| anti-human CD39 PE/Cy7 | Invitrogen | Cat#25-0399-42; RRID:AB_1582280 | |
| anti-human CD39 APC | BD Biosciences | Cat#560239; RRID:AB_1645459 | |
| anti-human CD39 PerCy7 | Biolegend | Cat#328212; RRID:AB_2099950 | |
| anti-human CD1c PE | Biolegend | Cat#331506; RRID:AB_1088999 | |
| anti-human CD19 APC/Fire750 | Biolegend | Cat#302258; RRID:AB_2629691 | |
| anti-human CD24 PE/AF610 | Invitrogen | Cat#MHCD2422; RRID:AB_10375871 | |
| anti-human CD24 PE/Dazzle594 | Biolegend | Cat#311134; RRID:AB_2566349 | |
| anti-human CD73 eFluor450 | Invitrogen | Cat#48-0739-42; RRID:AB_11041811 | |
| anti-human CD73 PE/Dazzle594 | Biolegend | Cat#344020; RRID:AB_2565301 | |
| anti-human IgD AF700 | Biolegend | Cat#348230; RRID:AB_2563335 | |
| anti-human IgM BV421 | Biolegend | Cat#314516; RRID:AB_2561443 | |
| anti-human CD38 PerCP | Biolegend | Cat#303520; RRID:AB_893313 | |
| anti-human CD38 FITC | Biolegend | Cat#303504; RRID:AB_314356 | |
| anti-human CD10 PE/Cy7 | Biolegend | Cat#312214; RRID:AB_2146548 | |
| anti-human CD23 APC | Invitrogen | Cat#17-0238-42; RRID:AB_2016666 | |
| anti-human CD45RB PE | Biolegend | Cat#310204; RRID:AB_314807 | |
| anti-human CD45 AmCyan | BD Bioscience | Cat#339192; RRID:AB_647361 | |
| anti-human CD27 APC | Invitrogen | Cat#17-0279-42; RRID:AB_10671130 | |
| anti-human CD27 PE/Cy5 | Invitrogen | Cat#15-0279-42; RRID:AB_10717249 | |
| anti-human CD21 FITC | Invitrogen | Cat#11-0219-42; RRID:AB_2572442 | |
| Rhodamine 123 | Invitrogen | Cat#R302 | |
| anti- CD3 (SP7) | abcam | Cat#ab16669; RRID:AB_443425 | |
| anti- CD20 (SP32) | abcam | Cat#ab64088; RRID:AB_1139386 | |
| anti- CD39 (EPR20627) | abcam | Cat#ab223842; RRID:AB_2889212 | |
| **Biological samples** | | | |
| Human PBMCs | Ulm University Medical Center | N/A | |
| Human tumor tissues | Ulm University Medical Center | N/A | |
| **Chemicals, peptides, and recombinant proteins** | | | |
| Ficoll-Paque | Cytvia | | Cat#17-440-02; RRID: |
| Erythrocyte lysis |  | |  |
| **Critical commercial assays** | | | |
| Tumor dissociation kit | Miltenyi Biotec | Cat#130-095-929; RRID: | |
| CD45 (TIL) MicroBeads, human | Miltenyi Biotec | Cat#130-118-780; RRID: | |
| Versacomp antibody capture bead kit |  | Cat#B22804; RRID: | |
| **Software and algorithms** | | | |
| Graphpad Prism v9 |  | RRID:SCR_002798 | |
| Kaluza |  | RRID:SCR_016182 | |
| FlowJo v10 | FlowJo LLC | RRID:SCR_008520 | |
| R version 4.2.0 |  | N/A | |
| R version 4.0.3 |  | N/A | |

# 2 Supplementary Figures

**Fig. S1 Gating strategy.** Gating strategies for MZB (**a**), MZP (**b**), and GCB (**c**).

**Fig. S2 Proportion and Absolute Number of MZB in Blood.** Proportion and absolute number of B cells (**a**) and MZBs (**b**) within blood samples, with stratified analysis based on HPV status (**c**), primary tumor location (**d**), T stage (**e**) and AJCC clinical stage (**f**).

**Fig. S3 Proportion and Absolute Number of MZP Cells in Blood.** Proportion and absolute number of B cells (**a**) and MZPs (**b**) within blood samples, with stratified analysis based on HPV status (**c**), primary tumor location (**d**), T stage (**e**) and AJCC clinical stage (**f**).

**Fig. S4 Proportion and Absolute Number of GCB Cells in Blood.** Proportion and absolute number of B cells (**a**) and GCBs (**b**) within blood samples, with stratified analysis based on HPV status (**c**), primary tumor location (**d**), T stage (**e**) and AJCC clinical stage (**f**).

**Fig. S5 B Cell Population Differences Between HPV^+^ and HPV^−^ Patients.** B cell subset distribution detected using the GCB panel in dissected HNSCC tumor samples. Comparative analysis highlights differences in the frequencies of CD38^hi^ antibody-secreting cells (**a**), CD38⁻IgD⁻ memory B cells (**b**), CD38^+^IgD^+^ activated B cells (**c**), CD38⁻IgD^+^ naive B cells (**d**), CD27⁻CD21^lo^ tissue-like memory B cells (**e**), and CD27⁺CD21⁺/⁻ resting/activated memory B cells (**f**) between HPV⁺ and HPV⁻ patients.

**Fig. S6 scRNA-seq Quality Control and Normalization.** (**a-b**) Quality control metrics for integrating four scRNA-seq datasets. (**c**) UMAP plots showing the distribution of cells labeled by donor identities, datasets, and tissue origin. (**d**) Kernel density plot of the MZP cluster, calculated using the gene set CD1C^+^, IGHD^+^, IGHM^+^, PTPRC (CD45)^+^, and ABCB1^+^.

**Fig. S7 Pathway Enrichment Analysis.** Comparison of enriched pathways between HPV^+^ and HPV^-^ HNSCC patients, annotated by ReactomePA database.

**Fig. S8 Cellular Communication of TIBs in HNSCC patients.** (**a**) CellChat-predicted significant ligand-receptor pair numbers between CD4^+^ cells, CD8^+^ T cells and B cell subsets in primary HNSCC tumor milieu. (**b**) Dot plot showing the L-R pairs and intensity between MZB-1 and MZB-2 clusters and other TILs.

**Fig. S9 Cell Communication Analysis between Healthy Donors (HD) and HNSCC.** (**a**) Signaling pathways and ligand-receptor (L-R) pairs involved in MHC-I and MHC-II associated signaling between MZB-1 and MZB-2 clusters and other TILs in HDs. (**b**) Dot plot showing the L-R pairs between MZB-1 and MZB-2 clusters and other TILs in HDs (red) and HNSCC patients (blue). (**c**) Comparison of signaling pathway intensities between HDs and HNSCC patients.

**Fig. S10** (a) CIBERSORTx deconvolution of scRNA-seq data to bulk RNA-seq TCGA HNSC data for constructing an immune infiltration matrix, showing TIL cell proportion of tumor tissues from HPV^+^ HNSCC patients (n = 75) compared to HPV^-^ counterparts (n = 494). (**b**) Gene Set Intersection shows gene set overlap among IgM-only, TS, and MZB-1 cells in HNSCC patients. (**c** ) CytoTRACE Pseudo-time Trajectory Analysis suggests a developmental link between the MZB-1 and MZB-2 clusters.

# 3 Supplementary Tables

**Table S1. Clinicopathologic characteristics of HNSCC patients in the MZB cohort (PBMCs)**

| **HNSCC patients** | N = 81 (PBMCs) |
| --- | --- |
| **Age (y), median (range)** | 66 (40 – 90) |
| **Sex** |  |
| Male | 63 (77.78%) |
| Female | 18 (22.22%) |
| **Tobacco use** | 57 (70.37%) |
| **Alcohol use** | 53 (65.43%) |
| **HPV status** |  |
| Positive | 24 (29.63%) |
| Negative | 20 (24.69%) |
| Undetermined | 37 (45.68%) |
| **Tumor site** |  |
| Oral cavitiy | 10 (12.34%) |
| Larynx/Hypopharynx | 20 (48.78%) |
| Nasopharynx/Oropharynx | 44 (54.32%) |
| Undetermined | 7 (8.64%) |
| **Clinical T classification** |  |
| T1 | 13 (16.04%) |
| T2 | 25 (30.86%) |
| T3 | 15 (18.52%) |
| T4 | 25 (30.86%) |
| TX | 3 (3.70%) |
| **Clinical N classification** |  |
| N0 | 29 (35.80%) |
| N1 | 18 (22.22%) |
| N2 | 20 (24.69%) |
| N3 | 9 (11.11%) |
| N4 | 5 (6.17%) |
| **Clinical M classification** |  |
| M0 | 70 (86.42%) |
| M1 | 5 (6.17%) |
| MX | 6 (7.41%) |
| **Clinical tumor stage** |  |
| Stage I | 8 (9.88%) |
| Stage II | 3 (3.70%) |
| Stage III | 15 (18.52%) |
| Stage IV | 55 (67.90%) |
| **Recurrence** | 16 (19.75%) |
| **Death** | 11 (13.58%) |

**Table S2. Clinicopathologic characteristics of healthy volunteers in the MZB cohort (PBMCs)**

| **Healthy volunteers** | N = 20 (PBMCs) |
| --- | --- |
| **Age (y), median (range)** | 57.28 (43 – 85) |
| **Sex** |  |
| Male | 16 (80%) |
| Female | 4 (20%) |
| **Tobacco use** | 12 (60%) |
| **Alcohol use** | 16 (80%) |

**Table S3. Clinicopathologic characteristics of HNSCC patients in the MZB cohort (tumor tissues)**

| **HNSCC patients** | N = 24 (TILs) |
| --- | --- |
| **Age(y), median (range)** | 54.46 (40 – 76) |
| **Sex** |  |
| Male | 20 (83.33%) |
| Female | 4 (16.67%) |
| **Tobacco use** | 18 (75%) |
| **Alcohol use** | 13 (54.17%) |
| **HPV status** |  |
| Positive | 5 (20.83%) |
| Negative | 8 (33.33%) |
| Undetermined | 11 (45.83%) |
| **Tumor site** |  |
| Oral cavitiy | 14 (58.33%) |
| Larynx/Hypopharynx | 3 (12.50%) |
| Nasopharynx/Oropharynx | 14 (58.33%) |
| **Pathological T classification** |  |
| T1 | 3 (12.50%) |
| T2 | 7 (29.17%) |
| T3 | 6 (25%) |
| T4 | 8 (33.33%) |
| **Pathological N classification** |  |
| N0 | 11 (45.83%) |
| N1 | 2 (8.33%) |
| N2 | 7 (29.17%) |
| N3 | 4 (16.67%) |
| **Pathological tumor stage** |  |
| Stage II | 5 (20.83%) |
| Stage III | 4 (16.67%) |
| Stage IV | 15 (62.5%) |

**Table S4. Clinicopathologic characteristics of HNSCC patients in the GCB cohort (PBMCs)**

| **HNSCC patients** | N = 42 (PBMCs) |
| --- | --- |
| **Age(y), median (range)** | 64.43 (45 – 85) |
| **Sex** |  |
| Male | 32 (76.19%) |
| Female | 11 (26.19%) |
| **Tobacco use** | 32 (76.19%) |
| **Alcohol use** | 31 (73.81%) |
| **HPV status** |  |
| Positive | 12 (28.57%) |
| Negative | 13 (30.95%) |
| Undetermined | 17 (40.48%) |
| **Tumor site** |  |
| Oral cavitiy | 6 (14.29%) |
| Larynx/Hypopharynx | 10 (23.81%) |
| Nasopharynx/Oropharynx | 22 (52.38%) |
| Undetermined | 4 (9.52%) |
| **Clinical T classification** |  |
| T1 | 7 (16.67%) |
| T2 | 16 (38.10%) |
| T3 | 7 (16.67%) |
| T4 | 10 (23.81%) |
| TX | 2 (4.76%) |
| **Clinical N classification** |  |
| N0 | 13 (30.95%) |
| N1 | 7 (16.67%) |
| N2 | 19 (45.23%) |
| N3 | 3 (7.14%) |
| **Clinical M classification** |  |
| M0 | 41 (97.62%) |
| M1 | 1 (2.38%) |
| **Clinical tumor stage** |  |
| Stage I | 6 (14.28%) |
| Stage II | 2 (4.76%) |
| Stage III | 8 (19.05%) |
| Stage IV | 26 (61.90%) |
| **Recurrence** | 12 (28.57%) |
| **Death** | 2 (4.76%) |

**Table S5. Clinicopathologic characteristics of healthy volunteers in the GCB cohort (PBMCs)**

| **Healthy volunteers** | N = 15 (PBMCs) |
| --- | --- |
| **Age (y), median (range)** | 53.27（43 – 72） |
| **Sex** |  |
| Male | 12 (80%) |
| Female | 3 (20%) |
| **Tobacco use** | 7 (46.67%) |
| **Alcohol use** | 11 (73.33%) |

**Table S6. Clinicopathologic characteristics of HNSCC patients in the GCB cohort (tumor tissues)**

| **HNSCC patients** | N = 16 (TILs) |
| --- | --- |
| **Age(y), median (range)** | 58 (40 – 72) |
| **Sex** |  |
| Male | 12 (75%) |
| Female | 4 (25%) |
| **Tobacco use** | 13 (81.25%) |
| **Alcohol use** | 14 (87.5%) |
| **HPV status** |  |
| Positive | 7 (43.75%) |
| Negative | 9 (56.25%) |
| **Tumor site** |  |
| Oral cavitiy | 6 (37.5%) |
| Larynx/Hypopharynx | 4 (25%) |
| Nasopharynx/Oropharynx | 6 (37.5%) |
| **Pathological T classification** |  |
| T1 | 3 (18.75%) |
| T2 | 5 (31.25%) |
| T3 | 4 (25%) |
| T4 | 3 (18.75%) |
| **Pathological N classification** |  |
| N0 | 6 (37.5%) |
| N1 | 2 (12.5%) |
| N2 | 5 (31.25%) |
| N3 | 3 (18.75%) |
| **Pathological tumor stage** |  |
| Stage II | 4 (25%) |
| Stage III | 4 (25%) |
| Stage IV | 7 (43.75%) |

**Table S7. Clinicopathologic characteristics of HNSCC patients in the MZP cohort (PBMCs)**

| **HNSCC patients** | N = 38 (PBMCs) |
| --- | --- |
| **Age(y), median (range)** | 59.85 (45 – 82) |
| **Sex** |  |
| Male | 29 (76.32%) |
| Female | 18 (47.37%) |
| **Tobacco use** | 28 (73.68%) |
| **Alcohol use** | 25 (65.79%) |
| **HPV status** |  |
| Positive | 11 (28.94%) |
| Negative | 12 (31.58%) |
| Undetermined | 15 (39.47%) |
| **Tumor site** |  |
| Oral cavitiy | 7 (18.42%) |
| Larynx/Hypopharynx | 7 (18.42%) |
| Nasopharynx/Oropharynx | 20 (52.63%) |
| Undetermined | 4 (8.64%) |
| **Clinical T classification** |  |
| T1 | 8 (21.05%) |
| T2 | 13 (34.21%) |
| T3 | 7 (18.42%) |
| T4 | 9 (23.68%) |
| TX | 1 (2.63%) |
| **Clinical N classification** |  |
| N0 | 14 (36.84%) |
| N1 | 7 (18.42%) |
| N2 | 14 (36.84%) |
| N3 | 3 (7.89%) |
| **Clinical M classification** |  |
| M0 | 34 (89.47%) |
| M1 | 2 (5.26%) |
| MX | 2 (5.26%) |
| **Clinical tumor stage** |  |
| Stage I | 5 (9.88%) |
| Stage II | 2 (3.70%) |
| Stage III | 10 (18.52%) |
| Stage IV | 21 (67.90%) |
| **Recurrence** | 16 (19.75%) |
| **Death** | 11 (13.58%) |

**Table S8. Clinicopathologic characteristics of healthy volunteers in the MZP cohort (PBMCs)**

| **Healthy volunteers** | N = 10 (PBMCs) |
| --- | --- |
| **Age (y), median (range)** | 48.75 (43 – 60) |
| **Sex** |  |
| Male | 8 (80%) |
| Female | 2 (20%) |
| **Tobacco use** | 6 (60%) |
| **Alcohol use** | 8 (80%) |

**Table S9. Clinicopathologic characteristics of HNSCC patients in the MZP cohort (tumor tissues)**

| **HNSCC patients** | N = 20 (TILs) |
| --- | --- |
| **Age(y), median (range)** | 62.6 (49 – 73) |
| **Sex** |  |
| Male | 16 (80%) |
| Female | 4 (20%) |
| **Tobacco use** | 12 (60%) |
| **Alcohol use** | 19 (95%) |
| **HPV status** |  |
| Positive | 5 (25%) |
| Negative | 6 (30%) |
| Undetermined | 9 (45%) |
| **Tumor site** |  |
| Oral cavitiy | 3 (15%) |
| Larynx/Hypopharynx | 7 (35%) |
| Nasopharynx/Oropharynx | 10 (50%) |
| **Pathological T classification** |  |
| T1 | 3 (15%) |
| T2 | 7 (45%) |
| T3 | 4 (20%) |
| T4 | 6 (30%) |
| **Pathological N classification** |  |
| N0 | 9 (45%) |
| N1 | 3 (15%) |
| N2 | 5 (25%) |
| N3 | 3 (15%) |
| **Pathological tumor stage** |  |
| Stage II | 5 (25%) |
| Stage III | 4 (20%) |
| Stage IV | 11 (55%) |

**Table S10. FACS panel for MZB cells**

| **Wave Band** | **Channel** | **Antibody** | **Dose** |
| --- | --- | --- | --- |
| **488 nm** | **FL1** | CD21 FITC | 3ul |
|  | **FL2** | CD1c PE | 5ul |
|  | **FL3** | CD73 PE/Dazzle594 | 4ul |
|  | **FL4** | CD27 PE/Cy5 | 5ul |
|  | **FL5** | CD39 Pe/Cy7 | 4ul |
| **633 nm** | **FL6** | CD23 APC | 4ul |
|  | **FL7** | IgD AF700 | 4ul |
|  | **FL8** | CD19 APC/Fire750 | 3ul |
| **405 nm** | **FL9** | IgM BV421 | 3ul |
|  | **FL10** | CD45 AmCyan | 3ul |

**Table S11. FACS panel for MZP cells**

| **Wave Band** | **Channel** | **Antibody** | **Dose** |
| --- | --- | --- | --- |
| **488 nm** | **FL1** | Rhodamine 123 | 6uM |
|  | **FL2** | CD45RB PE | 5ul |
|  | **FL3**  **FL4**  **FL5** | CD73 eFluor450  CD27 PE/Cy5  CD10 PE/Cy7 | 4ul  5ul  5ul |
| **633 nm** | **FL6**  **FL7**  **FL8** | CD39 APC  IgD AF700  CD19 APC/Fire750 | 4ul  4ul  3ul |
| **405 nm** | **FL9**  **FL10** | IgM BV421  CD45 AmCyan | 3ul  3ul |

**Table S12. FACS panel for GCB cells**

| **Wave Band** | **Channel** | **Antibody** | **Dose** |
| --- | --- | --- | --- |
| **488 nm** | **FL1** | CD38 FITC | 5ul |
|  | **FL3** | CD73 PE/Dazzle594 | 4ul |
|  | **FL4** | CD27 PE/Cy5 | 5ul |
|  | **FL5** | CD10 PE/Cy7 | 5ul |
| **633 nm** | **FL6** | CD39 APC | 4ul |
|  | **FL7** | IgD AF700 | 4ul |
|  | **FL8** | CD19 APC/Fire750 | 3ul |
| **405 nm** | **FL9** | IgM BV421 | 3ul |
|  | **FL10** | CD45 AmCyan | 3ul |
